# Effect of non-pharmacological interventions on depression in obese individuals: a network meta-analysis

**DOI:** 10.3389/fpsyt.2026.1715475

**Published:** 2026-02-16

**Authors:** Zhilin Chen, Ruiqing Wang, Yiwei Wang, Yangpu Zhang, Aiqun Song

**Affiliations:** 1 College of Acupuncture and Orthopedics, Hubei University of Chinese Medicine, Wuhan, Hubei, China; 2 Rehabilitation Medical Center, Xinhua Hospital of Hubei University of Chinese Medicine, Wuhan, Hubei, China; 3 Rehabilitation Medical Center, Hubei Provincial Hospital of Integrated Chinese and Western Medicine, Wuhan, Hubei, China; 4 Acupuncture Department, Hubei Provincial Hospital of Traditional Chinese Medicine, Wuhan, Hubei, China; 5 Hubei Sizhen Laboratory, Wuhan, Hubei, China; 6 Acupuncture Department, Affiliated Hospital of Hubei University of Chinese Medicine, Wuhan, Hubei, China; 7 Hubei Provincial Clinical Research Center for Acupuncture and Moxibustion in Obesity Treatment, Wuhan, Hubei, China

**Keywords:** cognitive behavioral therapy, depression, network meta-analysis, non-pharmacological interventions, obesity

## Abstract

**Background:**

Although the efficacy of non-pharmacological interventions for individuals with obesity and concurrent depression has been demonstrated, it remains unclear which strategy yields the most favorable outcomes. We conducted a network meta-analysis (NMA) to evaluate the comparative effectiveness of non-pharmacological interventions on depressive symptoms in overweight or obese individuals.

**Methods:**

Randomized controlled trials (RCTs) using non-pharmacological interventions were retrieved from Embase, PubMed, Cochrane Library, and Web of Science (up to March 2025). Standardized mean differences (SMD) were used for pooled analyses across depression scales, while mean differences (MD) were applied for single-scale analyses. Interventions were ranked using surface under the cumulative ranking curve (SUCRA) values. All statistical analyses were performed using R4.5.1 and Stata 15.

**Results:**

36 RCTs involving 11,153 participants were included, with 16 non-pharmacological interventions assessed by five depression scales. SUCRA analysis revealed that in the summary data, cognitive behavioral therapy (CBT) ranked higher (77.8%), while group meetings + telephone consultations (GM+TC) ranked lower (23.7%). For Beck Depression Inventory (BDI), CBT (94.9%) ranked higher, while behavioral therapy (BT) (34.4%) ranked lower. For the BDI-II, BT plus lifestyle intervention (BT+LI) (97.6%) ranked higher, while weight management and structured support programs (WMSSP) (2.6%) ranked lower. However, because the BDI-II network was sparse and relied on indirect comparisons, this ranking should be interpreted cautiously. For the Patient Health Questionnaire-9 (PHQ-9), CBT-based combined intervention (CBT-CI) ranked higher (97.9%), while dietary intervention ranked lower (10.2%). In the assessment using the Center for Epidemiologic Studies Depression Scale (CES-D), psychosocial and mind-body interventions (PMBI) (84.5%) ranked higher, while GM+TC (0.5%) ranked lower. For the Hospital Anxiety and Depression Scale (HADS), WMSSP (81.9%) had higher SUCRA values, while the control (24.7%) had lower values. Given the sparse, indirect network for HADS, the confidence in its high SUCRA ranking was limited.

**Conclusion:**

CBT appears effective, though overall certainty is limited by methodological concerns across many RCTs. BT+LI shows benefits in BDI-II assessments; PMBI shows effectiveness in CES-D evaluations. In the HADS network, WMSSP had high SUCRA values; however, intergroup differences were not statistically significant.

**Systematic Review Registration:**

https://www.crd.york.ac.uk/prospero/, identifier CRD420251022091.

## 1 Introduction

Obesity is an increasingly serious public health issue worldwide. It is not only a direct risk factor for multiple chronic diseases, such as diabetes and hypertension, but is also closely associated with numerous other health problems (1, 2). With socioeconomic development and changing lifestyles, it is projected that by 2035, over half of the global adult population will be affected by obesity, imposing a significant burden on society and healthcare resources (3). Epidemiological studies indicate that the incidence of depression is notably higher among obese individuals than normal-weight individuals (4, 5). Individuals with depression face heightened obesity risks due to metabolic disorders, psychosocial stress, and altered behavioral patterns (e.g., abnormal appetite, reduced physical activity) (6, 7). Although the association between obesity and depression has been extensively validated, intervention strategies for individuals with comorbid obesity and depression remain significantly challenging. While pharmacological treatments can partially alleviate symptoms, long-term use may bring metabolic side effects (8) and low patient compliance (9). Consequently, non-pharmacological interventions have emerged as a central focus in research and practice due to their high safety profiles, minimal side effects, and potential for multi-target regulation (10, 11).

Currently, there are diverse non-pharmacological interventions for depression in obese populations, including psychotherapy (e.g., cognitive behavioral therapy [CBT] and mindfulness training), lifestyle modifications (e.g., exercise and dietary management), digital health interventions (e.g., VR technology), and social support. These approaches can help patients adjust dietary and lifestyle habits, identify and modify negative thought patterns and behavioral routines through multiple pathways, thereby alleviating depressive symptoms and improving mood (12, 13). As demonstrated by Abdollahi et al. (14), CBT significantly improved obesity-related depressive symptoms by facilitating positive cognitive and behavioral shifts in patients. Other studies indicated that time-restricted eating and mindfulness-based intervention (MBI) demonstrated favorable outcomes in enhancing quality of life and palliating psychological burdens, bringing about positive life changes for patients (15, 16). Additionally, dietary and exercise interventions demonstrated favorable efficacy in improving physical conditions and gaining positive psychological feedback through scientifically guided nutrition and appropriate physical activity (17). However, existing research has primarily focused on single intervention measures, lacking direct comparisons and ranking of the efficacy of different intervention methods. Furthermore, network meta-analysis (NMA) studies on depression interventions for obese populations remain absent, making it difficult to comprehensively understand the relative effectiveness and applicability of various intervention approaches. Therefore, this study aims to systematically retrieve and integrate existing evidence to investigate the effectiveness of different non-pharmacological interventions in improving depressive symptoms among obese individuals.

The findings of this NMA will address the evidence gap in the interdisciplinary field of obesity and depression, providing clinicians with a tiered evidence-based framework for intervention selection and offering public health policymakers a scientific basis for optimizing resource allocation. By elucidating the mechanisms and appropriate application scenarios for non-pharmacological interventions, this NMA will also advance the development of personalized treatment strategies, ultimately improving the physical and mental health outcomes of obese populations.

## 2 Materials and methods

The present NMA was conducted according to PRISMA statements (18) and registered in the Prospero (registration number: CRD420251022091). Ethical approval was not required according to Health Research Authority guidance. Data are available on reasonable request.

### 2.1 Search strategy

A comprehensive search was conducted in PubMed, Embase, Cochrane Library, and Web of Science from the inception of each database to March 16, 2025. Search terms comprised both MeSH terms and free-text words, such as “Obesity”, “Overweight”, “Diet”, “Exercise”, “Cognitive Behavioral Therapy”, “Psychosocial Intervention”, “Social Support”, “Mindfulness-Based Stress Reduction”, “Exercise, Aerobic”, “Cognitive Behavioral Therapy”,”Psychosocial therapy”,”Psychotherapy”, and “Depression”. Boolean operators were used to combine MeSH terms and free-text words. Detailed information is provided in **Appendix 1**. Reference lists of included studies were manually searched, and citations from relevant reviews were traced to identify potentially overlooked studies.

### 2.2 Eligibility criteria

Inclusion criteria were as follows: randomized controlled trials (RCTs) meeting PICOS criteria. Study subjects were overweight or obese individuals meeting the latest WHO criteria. Although the target population was adults, one RCT enrolled adolescents (14.99 years). This age deviation was recorded and later addressed through sensitivity analyses. Intervention measures encompassed cognitive therapy (CT), behavioral therapy (BT), cognitive and behavioral interventions (CBI), Pilates, exercise, CBT, CBT-based combined intervention (CBT-CI), diet + exercise, weight management and structured support programs (WMSSP), diet, lifestyle intervention (LI), BT + LI, LI + mental health support (LI+MHS), psychosocial and mind-body interventions (PMBI), dietary combined intervention (DCI), group meetings + telephone consultations (GM + TC). In this network meta-analysis, control conditions were defined as comparators that did not involve structured psychological or behavioral therapeutic interventions targeting depression. These included usual care, waiting-list or delayed-treatment conditions, assessment-only controls, and minimal or background comparators (e.g., health education or support) as defined in the original trials. It should be noted that the specific form of the control condition varied across studies according to their original trial design and comparison framework. The outcome measures were scores on various depression assessment scales (Beck Depression Inventory [BDI], BDI-II, Patient Health Questionnaire-9 [PHQ-9], Hospital Anxiety and Depression Scale [HADS], Center for Epidemiologic Studies Depression Scale [CES-D]). PMBIs were classified as a single category because the included trials—behavioral activation, mindfulness-based intervention, and commercial provider programs—shared common therapeutic elements, such as structured group support, emotional regulation components, and behavior-change strategies, despite differences in formats. This consolidation facilitated network connectivity and consistent comparisons across interventions.

Exclusion criteria included: meta-analyses, reviews, case reports, age ineligibility, animal studies, non-English literature, non-compliant study interventions, conference abstracts, non-compliant study diseases, non-compliant study types, non-compliant study objectives, guidelines, letters, and commentaries.

### 2.3 Literature screening

Two researchers (Zhilin Chen and Aiqun Song) independently screened the literature based on titles and abstracts in EndNote, then assessed the full texts of the remaining studies to confirm their eligibility for inclusion. In cases of disagreement, a third researcher, Ruiqing Wang, made the final decision.

### 2.4 Data extraction

Two researchers (Zhilin Chen and Aiqun Song) independently extracted relevant data. In cases of disagreement, a third researcher, Ruiqing Wang, made the final decision. Extracted data included authors, publication year, study country or region, intervention, sample size, age, sex ratio, comorbidities, treatment duration, type of depression assessment scale, and mean ± standard deviation of baseline-to-endpoint differences for each scale.

### 2.5 Quality assessment

Two researchers (Zhilin Chen and Aiqun Song) independently assessed study quality and risk of bias using the RCT ROB assessment tool 2.0 (ROB2.0) (19, 20). A third researcher (Ruiqing Wang) assisted in resolving discrepancies during the process. The ROB 2.0 assessment items included randomization bias, bias to established interventions, bias to missing outcome data, bias to outcome measurement, and bias to selective reporting. Studies were categorized as low risk, some concern, or high risk.

### 2.6 Statistical analysis

Statistical analysis was performed using R 4.5.1 and Stata 15, employing Monte Carlo Markov Chain (MCMC) within the Bayesian framework of NMA. The model ran four chains comprising 5,000 annealing iterations and converged after a total of 20,000 simulation iterations. A random-effects Bayesian hierarchical model was applied. Treatment effects were assigned vague Normal(0, 10,000) priors, and the between-study standard deviation (τ) was given a weakly informative Half-Normal prior. In the gemtc package, a scaling factor of 2.5 was used for the heterogeneity prior, which defined the scale of the Half-Normal distribution, thus controlling the expected magnitude of between-study variability. The standardized mean difference (SMD) was used for pooled analyses combining multiple depression scales (BDI, BDI-II, PHQ-9, CES-D, HADS). Mean difference (MD) was used only for analyses involving a single scale, with its 95% confidence interval (CI) calculated. Publication bias was assessed using funnel plots. Heterogeneity among study results was quantified using I^2^, with values ranging from 0% to 100%. An I^2^ value of 0% indicated no heterogeneity, while higher values suggested greater heterogeneity. Loop-specific heterogeneity was additionally explored using τ², providing complementary information for network-level variability. Model convergence was assessed using the Gelman–Rubin diagnostic plot, requiring all R̂ values to approach 1.00. A random-effects model was used for evidence with substantial heterogeneity, and a fixed-effects model for evidence with low heterogeneity. Nodes in the network diagram represented individual interventions, with node size proportional to sample size. Line thickness indicated the number of direct comparative studies, while absent lines denoted indirect comparisons. SUCRA scores ranked all intervention efficacies, with higher SUCRA values indicating greater potential for reducing depression scores.

## 3 Results

### 3.1 Literature screening results

**Figure 1:**
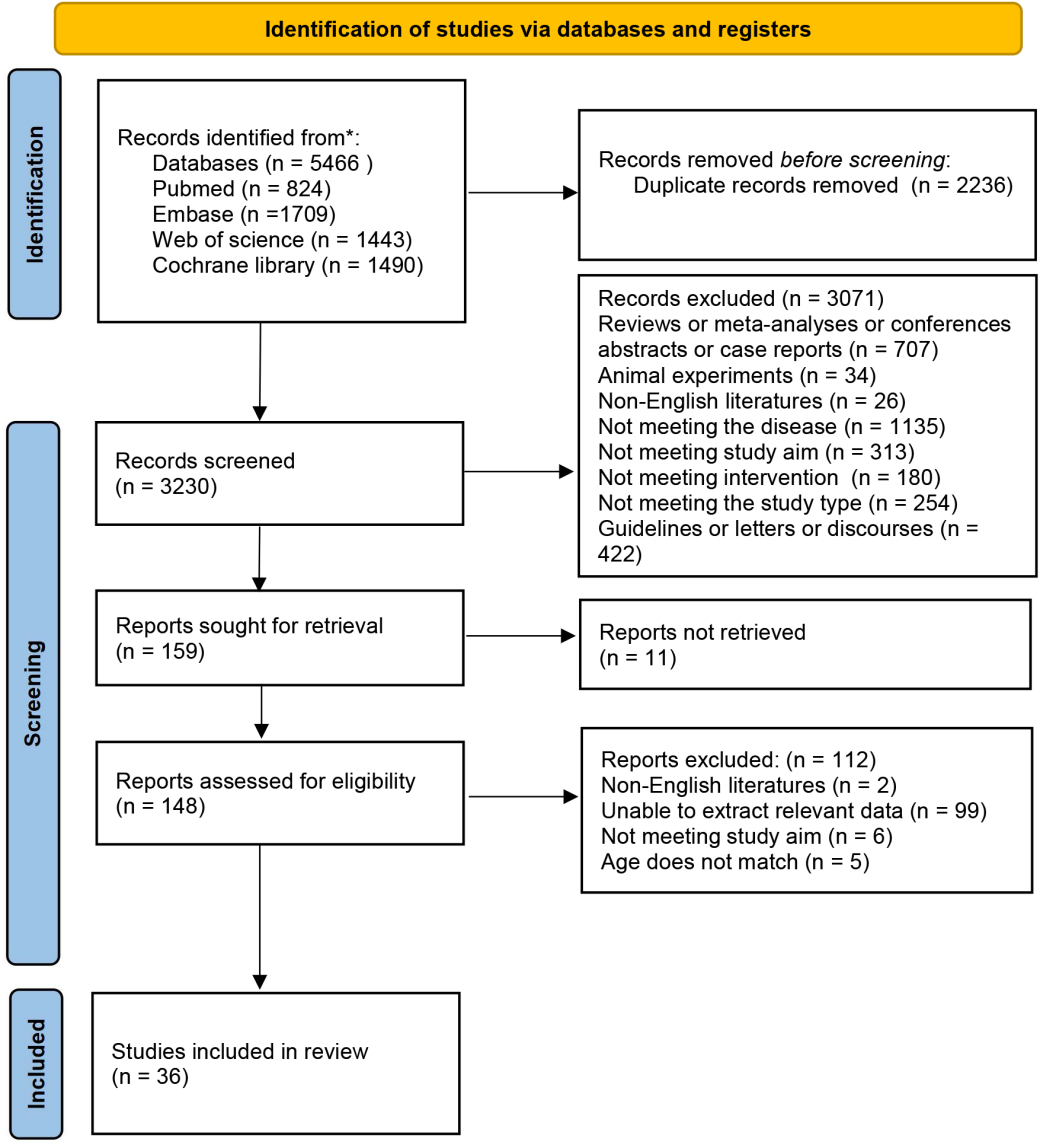
Literature screening process.

5,466 articles were retrieved. After excluding 2,236 duplicates, 3,230 articles remained. Following preliminary screening based on titles and abstracts, 159 articles were potentially eligible. After further full-text screening, 36 articles were ultimately included. The literature screening process is depicted in **Figure 1**.

### 3.2 Baseline data

The 36 studies included 11,153 patients, with ages ranging from 14.99 to 63.3 years. The proportion of female patients ranged from 0% to 100%. These studies spanned multiple regions, including North America, South America, Europe, Asia, and Oceania. Interventions included CT, BT, CBI, Pilates, exercise, CBT, CBT-CI, Diet + Exercise, WMSSP, diet, LI, BT + LI, LI + MHS, PMBI, DCI, and GM + TC. Baseline characteristics are outlined in **Table 1**.

**Table 1:** Baseline data.

Id	Author	Year	Country	Treatment	Sample	Age	Gender (percentage of female)	Complication	Treatment duration	Depression rating scale
1	Tanco	1998	Canada	Cognitive therapy(CT)	18	NR	100.00%	NR	8-week intervention + 6-month follow-up	BDI
				Behavioral therapy(BT)	19	NR	100.00%	NR	8-week intervention + 6-month follow-up	
				Control: usual care(routine clinical follow-up)	13	NR	100.00%	NR	8-week study period + 6-month follow-up	
2	Freitas	2018	Brazil	Weight Management and Structured Support Programs(WMSSP)	26	45.9 ± 7.7	96.15%	Moderate to severe asthma	3 months	HADS
				Control: usual control(sham procedures)	25	48.5 ± 9.6	100.00%	Moderate to severe asthma	3 months	
3	Xenaki	2018	Brazil	Cognitive and Behavioral Interventions(CBI)	22	46.91 ± 10.98	54.55%	NR	8 weeks	BDI
				Control: usual control(general health advice)	23	44.48 ± 10.12	47.83%	NR	8 weeks	
4	Werrij	2009	Netherlands	Cognitive therapy(CT)	96	44 ± 11.9	84.00%	NR	10 weeks	BDI
				Behavioral therapy(BT)	104	45 ± 12.2	78.00%	NR	10 weeks	
5	Vancini	2017	Brazil	Control:usual care(no-intervention control)	20	41.7 ± 12.6	60.00%	NR	8 weeks	BDI
				Aerobic exercise	21	42.4 ± 7.0	95.24%	NR	8 weeks	
				Pilates	22	55.9 ± 6.6	95.45%	NR	8 weeks	
6	Uemura	2019	Japan	Diet	22	62.0 ± 8.7	100.00%	NR	8 weeks	CES-D
				Control: usual care(no-intervention control)	22	63.3 ± 9.1	100.00%	NR	8 weeks	
7	Thomson	2010	Australia	Diet	30	29.3 ± 0.7	100.00%	polycystic ovary syndrome	20 weeks	CES-D
				Dietary Combined Intervention(DCI)	64	29.3 ± 0.7	100.00%	polycystic ovary syndrome	20 weeks	
8	Sockalingam	2022	Canada	Cognitive-Behavioral Therapy–Based Combined Intervention(CBT-CI)	40	47.68 ± 9.36	80.25%	After Bariatric Surgery	10 weeks	PHQ-9
				Control: usual control(standard bariatric care)	41	47.68 ± 9.36	80.25%	After Bariatric Surgery	10 weeks	
9	Sockalingam	2015	Canada	Cognitive-Behavioral Therapy–Based Combined Intervention(CBT-CI)	23	45.5 ± 8.9	83.00%	Before Bariatric Surgery	6 weeks	PHQ-9
				Control: usual control(standard pre-operative bariatric care)	24	45.5 ± 8.9	83.00%	Before Bariatric Surgery	6 weeks	
10	Shomaker	2019	United States of America	Psychosocial and Mind–Body Interventions(PMBI)	17	14.99 ± 1.69	100.00%	NR	6 weeks	CES-D
				Cognitive behavioral therapy(CBT)	15	14.99 ± 1.69	100.00%	NR	6 weeks	
11	Shomaker	2016	United States of America	Cognitive behavioral therapy(CBT)	61	NR	100.00%	NR	6 weeks	CES-D
				Control: usual control(non-therapeutic health education)	58	NR	100.00%	NR	6 weeks	
12	Shelley	2007	Canada	Cognitive behavioral therapy(CBT)	13	NR	100.00%	Binge eating behavior	6 weeks	BDI
				Control: delayed treatment	9	NR	100.00%	Binge eating behavior	3 weeks	
13	Seo	2023	South Korea	Exercise	47	48.4 ± 6.2	100.00%	NR	8 weeks	PHQ-9
				Control: usual care (no exercise intervention)	23	48.26 ± 7.56	100.00%	NR	8 weeks	
14	Sarsan	2006	Türkiye	Exercise	40	42.5 ± 8.9	100.00%	NR	12 weeks	BDI
				Control: usual care (no exercise intervention)	20	43.60 ± 6.46	100.00%	NR	12 weeks	
15	Painot	2001	Netherlands	Cognitive-Behavioral Therapy–Based Combined Intervention(CBT-CI)	25	42 ± 2.0	100.00%	NR	12 weeks	BDI, HADS
				Cognitive behavioral therapy(CBT)	35	44 ± 2.0	100.00%	NR	12 weeks	
16	Pagoto	2013	United States of America	Behavioral therapy+Lifestyle intervention(BT+LI)	78	45.6 ± 11.0	100.00%	NR	6 months	BDI-II
				Control: usual control(non-therapeutic health education)	83	46.2 ± 10.8	100.00%	NR	6 months	
17	Owens	2021	Netherlands, Spain, Germany, Britain	Cognitive and Behavioral Interventions(CBI)	513	47.5 ± 13.0	72.70%	NR	12 months	PHQ-9
				Control: placebo	256	47.54 ± 12.95	75.39%	NR	12 months	
				Diet	256	47.54 ± 12.95	80.47%	NR	12 months	
18	Berman	2015	United States of America	Cognitive and Behavioral Interventions(CBI)	9	52.7 ± 8.4	100.00%	NR	12 weeks	PHQ-9
				Control: active control(weight watchers)	10	50.3 ± 17.4	100.00%	NR	12 weeks	
19	Naparstek	2017	United States of America	Cognitive and Behavioral Interventions(CBI)	83	46.4 ± 12.0	83.33%	NR	3 months	CES-D
				Control: usual care(community campaign)	42	47.8 ± 10.5	80.72%	NR	3 months	
20	Moraes	2021	Brazil	Cognitive-Behavioral Therapy–Based Combined Intervention(CBT-CI)	31	35.98 ± 6.76	81.80%	NR	30 weeks	BDI
				Exercise	34	37.97 ± 5.96	82.40%	NR	30 weeks	
				Control: usual control(non-therapeutic health education)	33	36.18 ± 2.75	77.40%	NR	30 weeks	
21	Moncrieft	2016	United States of America	Lifestyle intervention(LI)	57	54.84 ± 8.27	64.90%	Type 2 Diabetes Mellitus	12 months	BDI-II
				Control: usual care (brief educational booklet)	54	54.78 ± 6.34	77.80%	Type 2 Diabetes Mellitus	12 months	
22	Levine	1996	United States of America	Exercise	44	36.3 ± 6.8	100.00%	Binge eating disorder	6 months	BDI
				Control: delayed treatment	33	37.0 ± 6.1	100.00%	Binge eating disorder	6 months	
23	Holland-Carter	2017	United States of America	Lifestyle intervention + Mental health support(LI+MHS)	279	55.2 ± 8.9	72.04%	Type 2 Diabetes Mellitus	12 months	PHQ-9
				Control: usual care(standard diabetes nutrition counseling)	284	54.9 ± 9.3	70.07%	Type 2 Diabetes Mellitus	12 months	
24	Heath	2022	Britain	Psychosocial and Mind–Body Interventions(PMBI)	1056	53.5 ± 13.7	67.81%	NR	52 weeks	HADS
				Control: usual care (brief self-help intervention)	211	51.9 ± 14.1	67.77%	NR	12 weeks	
25	Gade	2015	Norway	Cognitive behavioral therapy(CBT)	42	44.1 ± 9.8	64.30%	Before Bariatric Surgery	10 weeks	HADS
				Control: usual care (nutritional support and education)	38	41.2 ± 9.6	73.70%	Before Bariatric Surgery	10 weeks	
26	Faulconbridge	2012	United States of America	Diet+Exercise	2563	NR	NR	Type 2 Diabetes Mellitus	12 months	BDI
				Control: usual care(support and education)	2566	NR	NR	Type 2 Diabetes Mellitus	12 months	
27	Drew	2022	Australia	Lifestyle intervention + Mental health support(LI+MHS)	62	48.4 ± 11.7	0.00%	NR	3 months	PHQ-9
				Control: delayed treatment	63	48.4 ± 11.7	0.00%	NR	3 months	
28	Demark-Wahnefried	2015	United States of America	Group meetings + telephone consultations(GM+TC)	344	56.0 ± 9.47	100.00%	Breast Cancer survivors	24 months	CES-D
				Control: usual care(support and education)	348	56.4 ± 9.53	100.00%	Breast Cancer survivors	24 months	
29	Cassin	2016	Canada	Cognitive-Behavioral Therapy–Based Combined Intervention(CBT-CI)	23	45.5 ± 8.9	83.00%	NR	7 weeks	PHQ-9
				Control: usual care(standard preoperative care)	24	45.5 ± 8.9	83.00%	NR	7 weeks	
30	Bacon	2005	United States of America	Weight Management and Structured Support Programs(WMSSP)	19	41.4 ± 3.0	100.00%	NR	6 months	BDI
				Diet	19	40.0 ± 4.4	100.00%	NR	6 months	
31	Altazan	2019	United States of America	Weight Management and Structured Support Programs(WMSSP)	37	29.1 ± 4.4	100.00%	NR	6 months	BDI-II
				Control: usual care(usual obstetric care)	17	29.5 ± 5.1	100.00%	NR	6 months	
32	Alfonsson	2015	Sweden	Psychosocial and Mind–Body Interventions(PMBI)	50	45.50 ± 10.71	92.00%	Binge eating disorder(BED)	10 weeks	HADS
				Control: delayed treatment	46	44.17 ± 10.90	95.70%	Binge eating disorder(BED)	10 weeks	
33	Abdollahi	2018	Iran	Cognitive behavioral therapy(CBT)	37	28.44 ± 4.24	100.00%	polycystic ovary syndrome	8 weeks	BDI
				Control: usual care(routine clinical follow-up)	37	27.44 ± 4.6	100.00%	polycystic ovary syndrome	8 weeks	
34	Lin	2023	United States of America	Diet	60	44 ± 11	83.00%	NR	12 months	BDI-II
				Control: usual care(no-intervention control)	30	44 ± 13	80.00%	NR	12 months	
35	Kiernan	2001	United States of America	Diet + Exercise	39	38.5 ± 6.4	51.85%	NR	12 months	BDI
				Diet	40	38.5 ± 6.4	43.66%	NR	12 months	
				Control: usual care(no-intervention control)	40	38.5 ± 6.4	49.37%	NR	12 months	
36	Sanchez	2017	Canada	Diet	62	35.0 ± 10.0	100.00%	NR	24 weeks	BDI
				Control: placebo	63	37.0 ± 10.0	100.00%	NR	24 weeks	

NR, not reported.

### 3.3 Quality assessment

**Figure 2:**
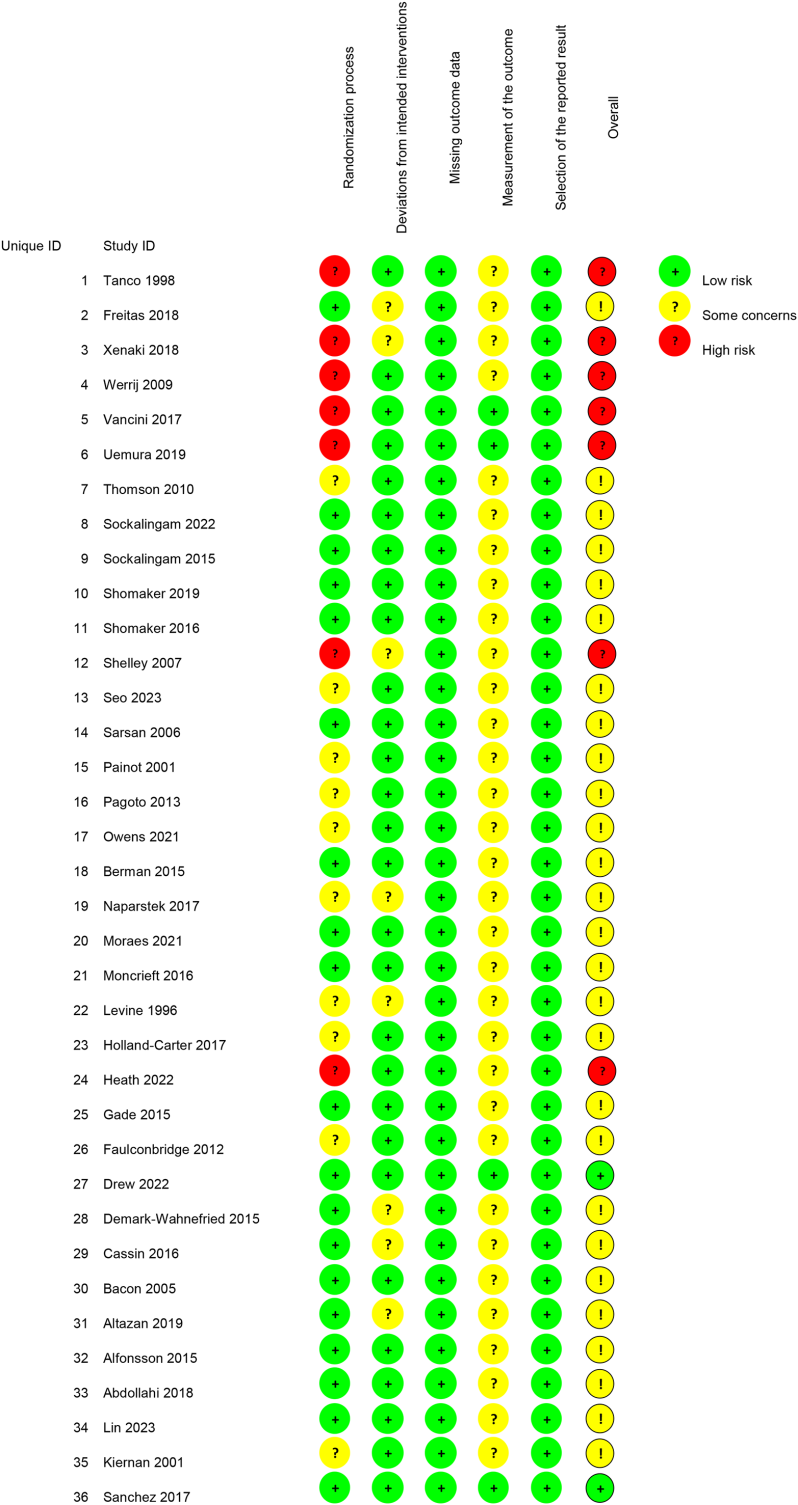
ROB 2.0 assessment.

Based on ROB 2.0 assessment, two studies—Drew 2022 and Sanchez 2017—were rated as low risk. Tanco 1998, Xenaki 2018, Werrij 2009, Vancini 2017, Uemura 2019, Shelley 2007, and Heath 2022 were rated as high risk. The remaining 27 RCTs were assessed as having a possible risk (**Figure 2**). All RCTs demonstrated low risk of bias in the selection of the reported outcome and missing outcome data categories. Regarding deviations from intended interventions, all RCTs reported low risk except for eight studies: Freitas 2018, Xenaki 2018, Shelley 2007, Naparstek 2017, Levine 1996, Demark-Wahnefried 2015, Cassin 2016, and Altazan 2019, which reported possible risk. In the measurement of the outcome category, four RCTs—Vancini 2017, Uemura 2019, Drew 2022, and Sanchez 2017—had low risk of bias, while the remaining studies had possible risk of bias. In the randomization process category, seven RCTs—Tanco 1998, Xenaki 2018, Werrij 2009, Vancini 2017, Uemura 2019, Shelley 2007, and Heath 2022—had high risk of bias; ten RCTs—Thomson 2010, Seo 2023, Painot 2001, Pagoto 2013, Owens 2021, Naparstek 2017, Levine 1996, Holland-Carter 2017, Faulconbridge 2012, and Kiernan 2001 had moderate risk, while the remainder had low risk.

### 3.4 Evidence network diagrams

**Figure 3:**
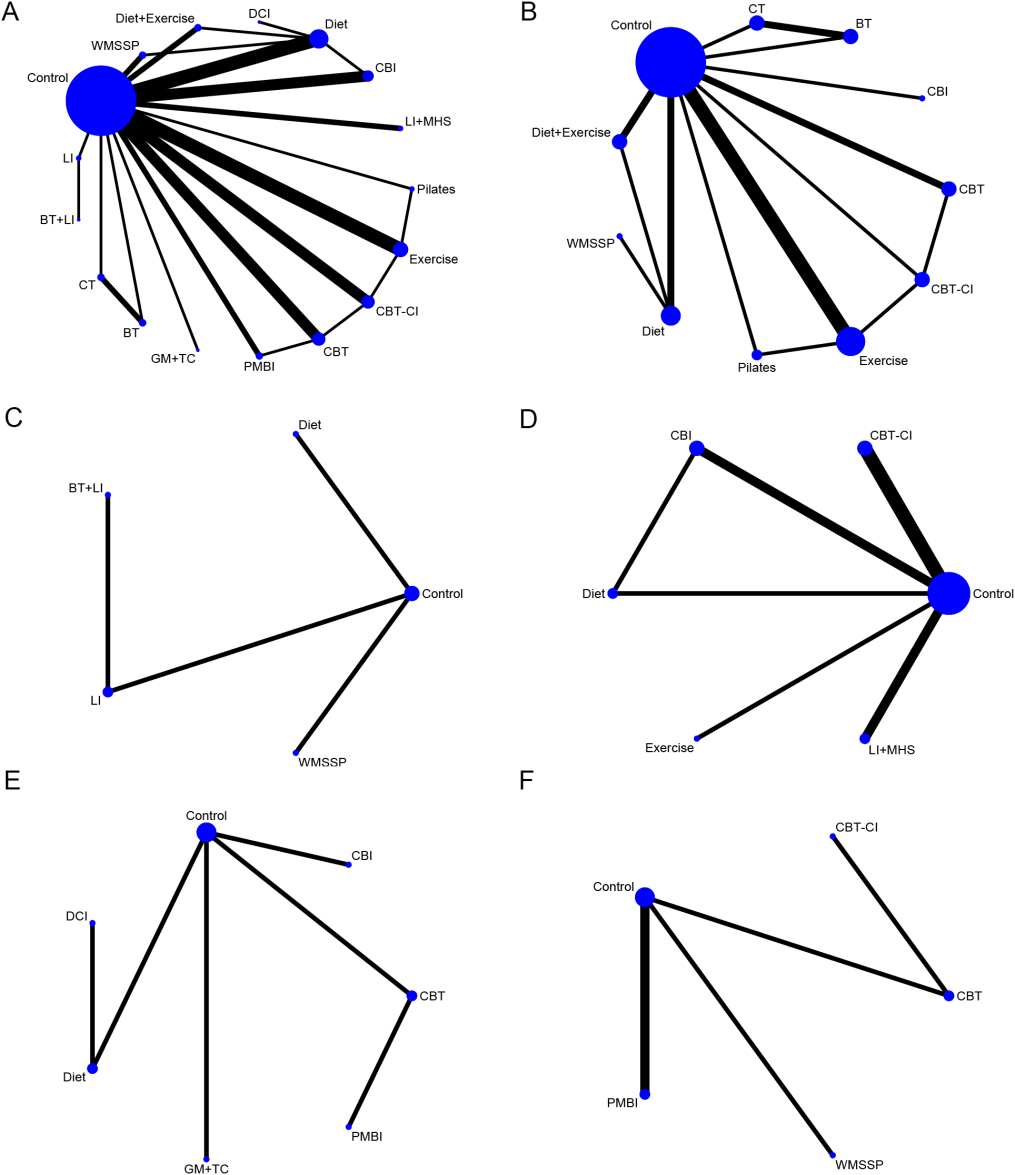
Network diagram. **(A)** Network Diagram of Summary Data; **(B)** Network Diagram of BDI Results; **(C)** Network Diagram of BDI-II Results; **(D)** Network Diagram of PHQ-9 Results; **(E)** Network Diagram of CES-D Results; **(F)** Network Diagram of HADS Results. BDI, Beck Depression Inventory; PHQ-9, Patient Health Questionnaire-9; CES-D, Center for Epidemiologic Studies Depression Scale.

**Figures 3A–F** present five distinct evidence networks for BDI, BDI-II, PHQ-9, CES-D, and HADS outcomes. The integrated data illustrated that all interventions were compared against the Control group. Diet was directly linked to derivative types, such as DCI and Diet + Exercise, forming a dense network centered around diet. Connections also existed with WMSSP, reflecting synergistic effects between the two. CBT and its derivative types formed a closed loop and had close connections with exercise-based interventions (Exercise, Pilates). Additionally, a direct link existed between CBT-CI and PMBI, suggesting continuity in their intervention pathways. Exercise-based interventions form a closed loop in the BDI network diagram, while Diet and Diet + Exercise also created a closed loop. Direct connections existed between these two major closed loops of CBT-based and exercise-based interventions, forming a densely connected network centered around Control. Additionally, relatively tight closed loops were observed for CT and BT. In the BDI-II network diagram, no closed loops formed. LI and Diet each established direct connections with Control, while BT + LI formed an indirect link to the overall network via LI. WMSSP remained isolated from other nodes, without connections to any interventions. In the PHQ-9 network diagram, CBI formed a direct connection with Diet and created a closed loop, while all other interventions established indirect links through Control. In the CES-D network diagram, PMBI was linked to Control via CBT, though both measures originated from the same source. The relationship between Diet and DCI followed the same pattern. In the HADS network diagram, PMBI was closely tied to Control, with all interventions except CBT-CI forming direct comparisons with Control.

### 3.5 Outcomes

The league table suggested that compared with Control, CBT considerably reduced depression scores (SMD: -5.51, 95% CI: -9.76 to -1.38), followed by CBT-CI (SMD: -4.97, 95% CI: -9.19 to -0.78), while the remaining interventions showed no measurable effect. Details are presented in **Table 2**.

**Table 2:** League table for summary data.

No.	X1	X2	X3	X4	X5	X6	X7	X8	X9	X10	X11	X12	X13	X14	X15	X16	X17
1	1	-1.75 (-11.65, 8.12)	-0.62 (-10.45, 9.17)	-3.54 (-8.09, 0.96)	-0.67 (-9.39, 8.1)	-0.87 (-5.05, 3.36)	-5.51 (-9.76, -1.38)	-4.97 (-9.19, -0.78)	-1.08 (-7.06, 4.92)	-2.03 (-7.78, 3.61)	-1.87 (-5.69, 1.92)	-4.2 (-13.77, 5.35)	-6.92 (-20.22, 6.38)	-1.38 (-7.78, 5)	-4.42 (-10.11, 1.02)	-2.79 (-14.2, 8.61)	1.57 (-7.39, 10.6)
2	1.75 (-8.12, 11.65)	2	1.15 (-5.7, 8.12)	-1.8 (-12.64, 9.08)	1.05 (-12.05, 14.35)	0.89 (-9.8, 11.69)	-3.75 (-14.49, 6.86)	-3.19 (-13.94, 7.52)	0.67 (-10.85, 12.2)	-0.28 (-11.6, 11.11)	-0.11 (-10.73, 10.46)	-2.45 (-16.18, 11.4)	-5.14 (-21.75, 11.46)	0.39 (-11.39, 12.22)	-2.67 (-14.06, 8.6)	-1.03 (-16.11, 13.99)	3.37 (-10.07, 16.6)
3	0.62 (-9.17, 10.45)	-1.15 (-8.12, 5.7)	3	-2.96 (-13.7, 7.91)	-0.12 (-13.09, 13.1)	-0.29 (-10.88, 10.46)	-4.9 (-15.67, 5.71)	-4.34 (-15.02, 6.31)	-0.5 (-11.91, 11.03)	-1.44 (-12.73, 9.87)	-1.3 (-11.81, 9.19)	-3.61 (-17.22, 10.04)	-6.31 (-22.87, 10.13)	-0.77 (-12.48, 10.88)	-3.84 (-15.13, 7.39)	-2.21 (-17.15, 12.77)	2.22 (-10.99, 15.41)
4	3.54 (-0.96, 8.09)	1.8 (-9.08, 12.64)	2.96 (-7.91, 13.7)	4	2.86 (-6.94, 12.73)	2.68 (-3.44, 8.89)	-1.96 (-8.18, 4.1)	-1.43 (-7.6, 4.78)	2.46 (-4.94, 9.91)	1.51 (-5.73, 8.67)	1.67 (-3.87, 7.16)	-0.64 (-11.18, 9.89)	-3.39 (-17.4, 10.7)	2.16 (-5.67, 10.01)	-0.88 (-8.18, 6.14)	0.76 (-11.33, 12.8)	5.12 (-4.91, 15.18)
5	0.67 (-8.1, 9.39)	-1.05 (-14.35, 12.05)	0.12 (-13.1, 13.09)	-2.86 (-12.73, 6.94)	5	-0.19 (-8.91, 8.57)	-4.85 (-14.57, 4.82)	-4.28 (-13.9, 5.3)	-0.41 (-10.98, 10.12)	-1.36 (-11.85, 9.13)	-1.19 (-10.75, 8.3)	-3.55 (-16.45, 9.5)	-6.28 (-22.1, 9.72)	-0.71 (-11.56, 10.06)	-3.75 (-14.18, 6.48)	-2.11 (-16.51, 12.26)	2.28 (-10.18, 14.81)
6	0.87 (-3.36, 5.05)	-0.89 (-11.69, 9.8)	0.29 (-10.46, 10.88)	-2.68 (-8.89, 3.44)	0.19 (-8.57, 8.91)	6	-4.63 (-10.6, 1.13)	-4.1 (-9.72, 1.47)	-0.21 (-7.52, 7.04)	-1.17 (-8.33, 5.88)	-1.01 (-6.68, 4.63)	-3.34 (-13.74, 7.06)	-6.06 (-20.04, 7.82)	-0.51 (-8.2, 7.06)	-3.56 (-10.65, 3.23)	-1.9 (-14.12, 10.16)	2.45 (-7.54, 12.29)
7	5.51 (1.38, 9.76)	3.75 (-6.86, 14.49)	4.9 (-5.71, 15.67)	1.96 (-4.1, 8.18)	4.85 (-4.82, 14.57)	4.63 (-1.13, 10.6)	7	0.54 (-4.82, 6.04)	4.42 (-2.85, 11.84)	3.47 (-3.55, 10.6)	3.64 (-1.97, 9.36)	1.31 (-9.02, 11.8)	-1.41 (-15.3, 12.69)	4.12 (-3.42, 11.86)	1.08 (-5.12, 7.19)	2.75 (-9.46, 14.94)	7.09 (-2.77, 17.13)
8	4.97 (0.78, 9.19)	3.19 (-7.52, 13.94)	4.34 (-6.31, 15.02)	1.43 (-4.78, 7.6)	4.28 (-5.3, 13.9)	4.1 (-1.47, 9.72)	-0.54 (-6.04, 4.82)	8	3.88 (-3.37, 11.26)	2.9 (-4.17, 10.04)	3.09 (-2.58, 8.77)	0.76 (-9.7, 11.21)	-1.96 (-15.86, 12.07)	3.57 (-4.04, 11.29)	0.54 (-6.39, 7.26)	2.18 (-10.04, 14.4)	6.54 (-3.33, 16.53)
9	1.08 (-4.92, 7.06)	-0.67 (-12.2, 10.85)	0.5 (-11.03, 11.91)	-2.46 (-9.91, 4.94)	0.41 (-10.12, 10.98)	0.21 (-7.04, 7.52)	-4.42 (-11.84, 2.85)	-3.88 (-11.26, 3.37)	9	-0.95 (-9.16, 7.15)	-0.8 (-7.26, 5.72)	-3.12 (-14.44, 8.1)	-5.83 (-20.41, 8.74)	-0.31 (-9.08, 8.42)	-3.34 (-11.61, 4.66)	-1.7 (-14.41, 10.82)	2.67 (-8.07, 13.54)
10	2.03 (-3.61, 7.78)	0.28 (-11.11, 11.6)	1.44 (-9.87, 12.73)	-1.51 (-8.67, 5.73)	1.36 (-9.13, 11.85)	1.17 (-5.88, 8.33)	-3.47 (-10.6, 3.55)	-2.9 (-10.04, 4.17)	0.95 (-7.15, 9.16)	10	0.16 (-6.02, 6.38)	-2.15 (-13.32, 9.02)	-4.87 (-19.28, 9.66)	0.64 (-7.89, 9.26)	-2.37 (-10.43, 5.45)	-0.75 (-13.2, 11.63)	3.62 (-6.97, 14.33)
11	1.87 (-1.92, 5.69)	0.11 (-10.46, 10.73)	1.3 (-9.19, 11.81)	-1.67 (-7.16, 3.87)	1.19 (-8.3, 10.75)	1.01 (-4.63, 6.68)	-3.64 (-9.36, 1.97)	-3.09 (-8.77, 2.58)	0.8 (-5.72, 7.26)	-0.16 (-6.38, 6.02)	11	-2.31 (-12.59, 7.88)	-5.03 (-18.81, 8.75)	0.48 (-6.92, 7.99)	-2.54 (-9.41, 4.06)	-0.92 (-11.74, 9.88)	3.45 (-6.3, 13.29)
12	4.2 (-5.35, 13.77)	2.45 (-11.4, 16.18)	3.61 (-10.04, 17.22)	0.64 (-9.89, 11.18)	3.55 (-9.5, 16.45)	3.34 (-7.06, 13.74)	-1.31 (-11.8, 9.02)	-0.76 (-11.21, 9.7)	3.12 (-8.1, 14.44)	2.15 (-9.02, 13.32)	2.31 (-7.88, 12.59)	12	-2.72 (-12.09, 6.6)	2.82 (-8.66, 14.35)	-0.23 (-11.37, 10.65)	1.41 (-13.43, 16.2)	5.83 (-7.27, 18.91)
13	6.92 (-6.38, 20.22)	5.14 (-11.46, 21.75)	6.31 (-10.13, 22.87)	3.39 (-10.7, 17.4)	6.28 (-9.72, 22.1)	6.06 (-7.82, 20.04)	1.41 (-12.69, 15.3)	1.96 (-12.07, 15.86)	5.83 (-8.74, 20.41)	4.87 (-9.66, 19.28)	5.03 (-8.75, 18.81)	2.72 (-6.6, 12.09)	13	5.54 (-9.2, 20.39)	2.48 (-12.09, 16.73)	4.14 (-13.34, 21.47)	8.5 (-7.44, 24.6)
14	1.38 (-5, 7.78)	-0.39 (-12.22, 11.39)	0.77 (-10.88, 12.48)	-2.16 (-10.01, 5.67)	0.71 (-10.06, 11.56)	0.51 (-7.06, 8.2)	-4.12 (-11.86, 3.42)	-3.57 (-11.29, 4.04)	0.31 (-8.42, 9.08)	-0.64 (-9.26, 7.89)	-0.48 (-7.99, 6.92)	-2.82 (-14.35, 8.66)	-5.54 (-20.39, 9.2)	14	-3.04 (-11.65, 5.31)	-1.37 (-14.6, 11.62)	2.97 (-8.03, 14.04)
15	4.42 (-1.02, 10.11)	2.67 (-8.6, 14.06)	3.84 (-7.39, 15.13)	0.88 (-6.14, 8.18)	3.75 (-6.48, 14.18)	3.56 (-3.23, 10.65)	-1.08 (-7.19, 5.12)	-0.54 (-7.26, 6.39)	3.34 (-4.66, 11.61)	2.37 (-5.45, 10.43)	2.54 (-4.06, 9.41)	0.23 (-10.65, 11.37)	-2.48 (-16.73, 12.09)	3.04 (-5.31, 11.65)	15	1.64 (-11.05, 14.4)	6.03 (-4.5, 16.74)
16	2.79 (-8.61, 14.2)	1.03 (-13.99, 16.11)	2.21 (-12.77, 17.15)	-0.76 (-12.8, 11.33)	2.11 (-12.26, 16.51)	1.9 (-10.16, 14.12)	-2.75 (-14.94, 9.46)	-2.18 (-14.4, 10.04)	1.7 (-10.82, 14.41)	0.75 (-11.63, 13.2)	0.92 (-9.88, 11.74)	-1.41 (-16.2, 13.43)	-4.14 (-21.47, 13.34)	1.37 (-11.62, 14.6)	-1.64 (-14.4, 11.05)	16	4.39 (-10.06, 18.89)
17	-1.57 (-10.6, 7.39)	-3.37 (-16.6, 10.07)	-2.22 (-15.41, 10.99)	-5.12 (-15.18, 4.91)	-2.28 (-14.81, 10.18)	-2.45 (-12.29, 7.54)	-7.09 (-17.13, 2.77)	-6.54 (-16.53, 3.33)	-2.67 (-13.54, 8.07)	-3.62 (-14.33, 6.97)	-3.45 (-13.29, 6.3)	-5.83 (-18.91, 7.27)	-8.5 (-24.6, 7.44)	-2.97 (-14.04, 8.03)	-6.03 (-16.74, 4.5)	-4.39 (-18.89, 10.06)	17

**Figure 4:**
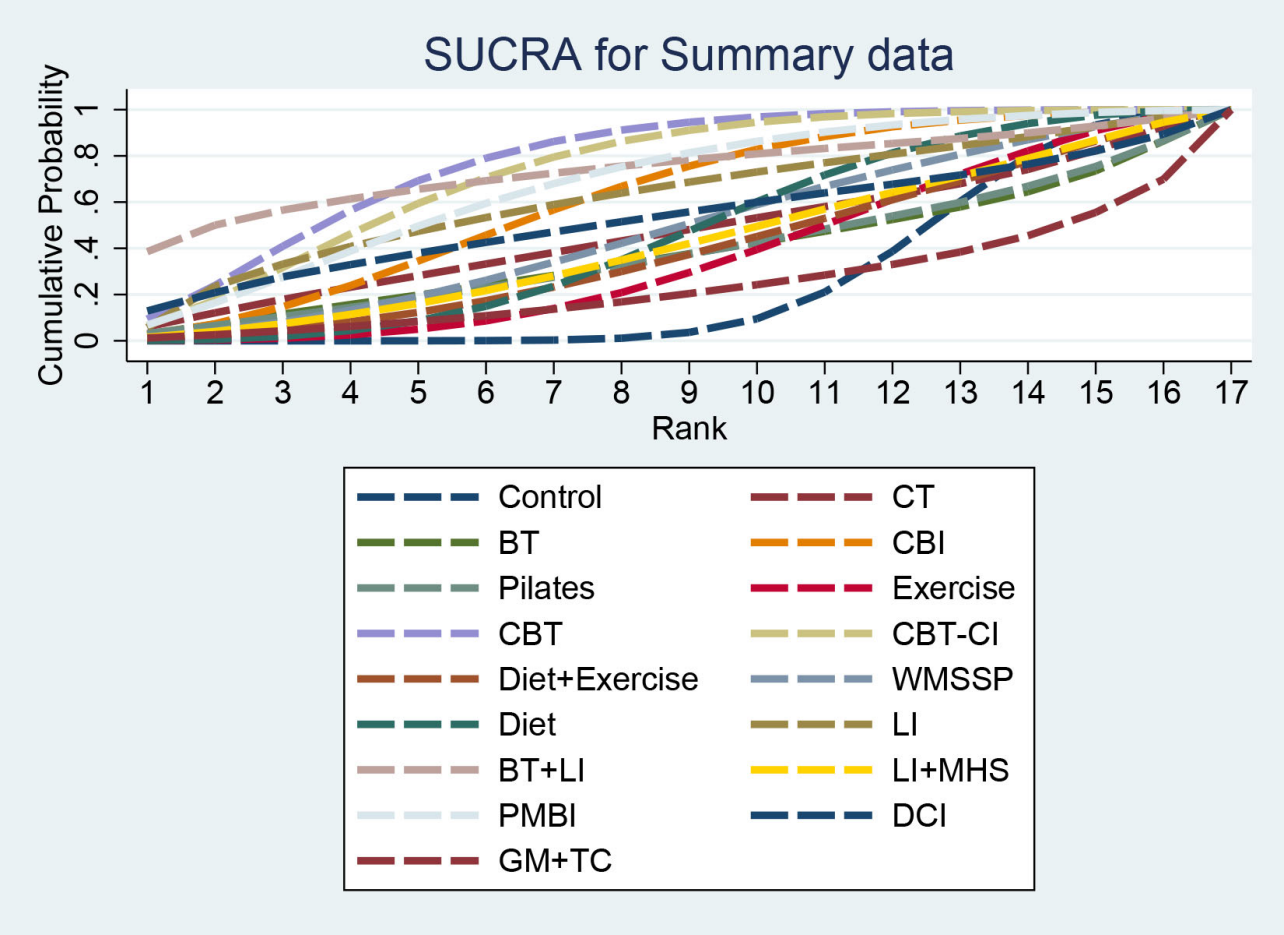
SUCRA of summary data. SUCRA, surface under the cumulative ranking curve.

SUCRA results indicated that CBT (77.8%) had a higher ranking probability, followed by BT+LI (74.0%) and CBT-CI (73.6%), while GM+TC (23.7%) had a lower ranking probability (**Figure 4**).

#### 3.5.1 BDI

Compared to Control, CBT markedly reduced depression scores (MD: -13.99, 95% CI: -21.11, -6.7), followed by CBT-CI (MD: -10.92, 95% CI: -18.99, -2.82). Compared to CBT, Diet + Exercise yielded inferior outcomes (MD: 13.19, 95% CI: 2.89, 23.25) (**Table 3**).

**Table 3:** League table for BDI.

No.	X1	X2	X3	X4	X5	X6	X7	X8	X9	X10	X11
1	1	-1.75 (-13.07, 9.03)	-0.62 (-11.74, 10.36)	-6.37 (-16.96, 4.26)	-0.97 (-11.08, 9.04)	-1.5 (-6.84, 4.06)	-13.99 (-21.11, -6.7)	-10.92 (-18.99, -2.82)	-0.81 (-8, 6.35)	-3.78 (-17.14, 9.78)	-0.96 (-8.12, 6.31)
2	1.75 (-9.03, 13.07)	2	1.13 (-6.64, 9.21)	-4.63 (-19.65, 11.02)	0.8 (-13.96, 15.88)	0.27 (-11.79, 12.99)	-12.24 (-25.13, 1.21)	-9.15 (-22.56, 4.72)	0.94 (-11.93, 14.3)	-2.02 (-19.15, 15.65)	0.81 (-12.16, 14.29)
3	0.62 (-10.36, 11.74)	-1.13 (-9.21, 6.64)	3	-5.76 (-20.78, 9.71)	-0.34 (-15.21, 14.56)	-0.88 (-13.04, 11.67)	-13.35 (-26.38, 0.01)	-10.29 (-23.88, 3.43)	-0.18 (-13.45, 13)	-3.13 (-20.54, 14.29)	-0.32 (-13.42, 12.94)
4	6.37 (-4.26, 16.96)	4.63 (-11.02, 19.65)	5.76 (-9.71, 20.78)	4	5.41 (-9.26, 19.91)	4.88 (-7.06, 16.83)	-7.64 (-20.4, 5.18)	-4.53 (-17.91, 8.67)	5.56 (-7.3, 18.25)	2.57 (-14.48, 19.75)	5.4 (-7.36, 18.28)
5	0.97 (-9.04, 11.08)	-0.8 (-15.88, 13.96)	0.34 (-14.56, 15.21)	-5.41 (-19.91, 9.26)	5	-0.52 (-10.45, 9.64)	-12.99 (-25.14, -0.71)	-9.92 (-22.44, 2.6)	0.16 (-12.11, 12.55)	-2.82 (-19.65, 14.1)	0.03 (-12.22, 12.47)
6	1.5 (-4.06, 6.84)	-0.27 (-12.99, 11.79)	0.88 (-11.67, 13.04)	-4.88 (-16.83, 7.06)	0.52 (-9.64, 10.45)	6	-12.46 (-21.18, -3.89)	-9.42 (-18.18, -0.82)	0.68 (-8.35, 9.62)	-2.28 (-16.88, 12.26)	0.54 (-8.53, 9.45)
7	13.99 (6.7, 21.11)	12.24 (-1.21, 25.13)	13.35 (-0.01, 26.38)	7.64 (-5.18, 20.4)	12.99 (0.71, 25.14)	12.46 (3.89, 21.18)	7	3.08 (-5.61, 11.56)	13.19 (2.89, 23.25)	10.2 (-5.16, 25.58)	13.04 (2.74, 23.25)
8	10.92 (2.82, 18.99)	9.15 (-4.72, 22.56)	10.29 (-3.43, 23.88)	4.53 (-8.67, 17.91)	9.92 (-2.6, 22.44)	9.42 (0.82, 18.18)	-3.08 (-11.56, 5.61)	8	10.12 (-0.64, 20.92)	7.13 (-8.5, 23.03)	9.96 (-0.84, 20.81)
9	0.81 (-6.35, 8)	-0.94 (-14.3, 11.93)	0.18 (-13, 13.45)	-5.56 (-18.25, 7.3)	-0.16 (-12.55, 12.11)	-0.68 (-9.62, 8.35)	-13.19 (-23.25, -2.89)	-10.12 (-20.92, 0.64)	9	-2.97 (-17.12, 11.36)	-0.15 (-8.68, 8.51)
10	3.78 (-9.78, 17.14)	2.02 (-15.65, 19.15)	3.13 (-14.29, 20.54)	-2.57 (-19.75, 14.48)	2.82 (-14.1, 19.65)	2.28 (-12.26, 16.88)	-10.2 (-25.58, 5.16)	-7.13 (-23.03, 8.5)	2.97 (-11.36, 17.12)	10	2.83 (-8.69, 14.27)
11	0.96 (-6.31, 8.12)	-0.81 (-14.29, 12.16)	0.32 (-12.94, 13.42)	-5.4 (-18.28, 7.36)	-0.03 (-12.47, 12.22)	-0.54 (-9.45, 8.53)	-13.04 (-23.25, -2.74)	-9.96 (-20.81, 0.84)	0.15 (-8.51, 8.68)	-2.83 (-14.27, 8.69)	11

**Figure 5:**
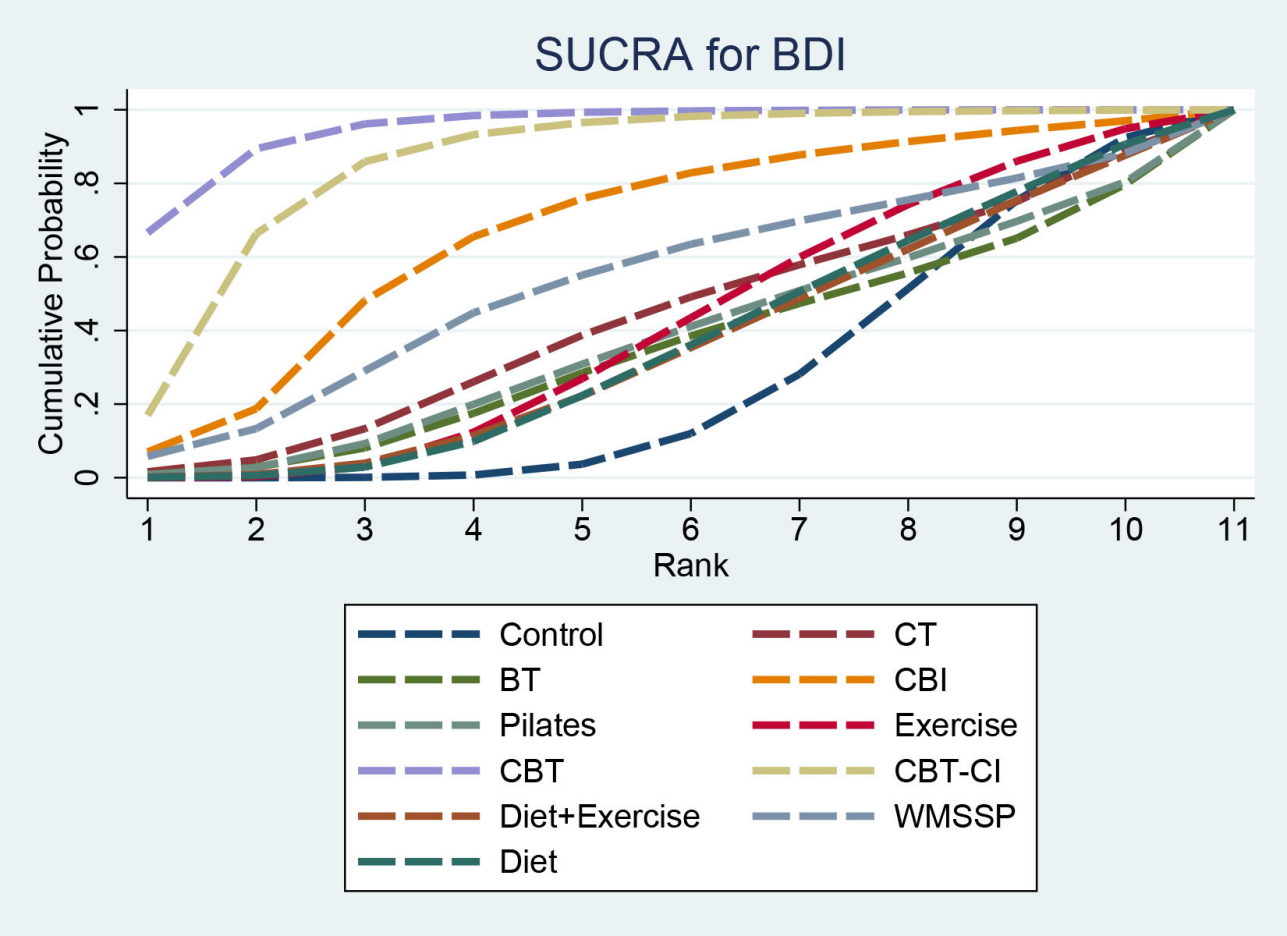
SUCRA for BDI. SUCRA, surface under the cumulative ranking curve; BDI, Beck Depression Inventory.

SUCRA results indicated that CBT (94.9%) had the highest probability of being among the top-ranked interventions, followed by CBT-CI (85.5%) and CBI (66.7%). BT (34.4%) had a lower ranking probability (**Figure 5**).

#### 3.5.2 BDI-II

Compared with Control, BT+LI lowered depression scores (MD: -6.91, 95% CI: -11.16, -2.68), followed by LI (MD: -4.2, 95% CI: -7.53, -0.91). Compared with WMSSP, Diet also demonstrated favorable effects (MD: -5.26, 95% CI: -10.34, -0.22) (**Table 4**).

**Table 4:** League table for BDI-II.

No.	X1	X2	X3	X4	X5
1	1	-4.2 (-7.53, -0.91)	-6.91 (-11.16, -2.68)	2.51 (-0.95, 6.02)	-2.75 (-6.43, 0.89)
2	4.2 (0.91, 7.53)	2	-2.7 (-5.33, -0.07)	6.72 (1.9, 11.55)	1.48 (-3.53, 6.39)
3	6.91 (2.68, 11.16)	2.7 (0.07, 5.33)	3	9.42 (3.95, 14.93)	4.17 (-1.48, 9.76)
4	-2.51 (-6.02, 0.95)	-6.72 (-11.55, -1.9)	-9.42 (-14.93, -3.95)	4	-5.26 (-10.34, -0.22)
5	2.75 (-0.89, 6.43)	-1.48 (-6.39, 3.53)	-4.17 (-9.76, 1.48)	5.26 (0.22, 10.34)	5

**Figure 6:**
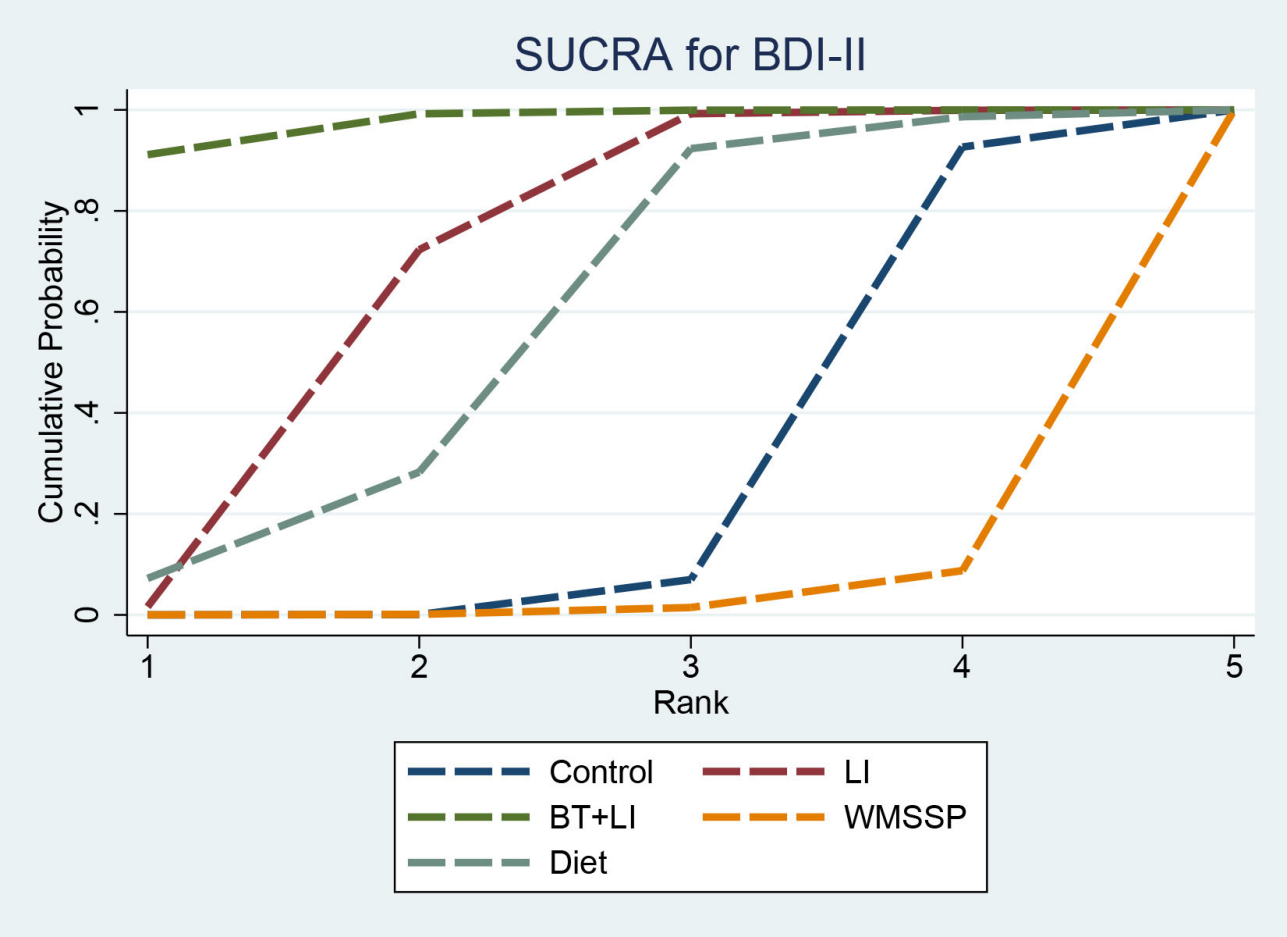
SUCRA for BDI-II. SUCRA, surface under the cumulative ranking curve; BDI, Beck Depression Inventory.

SUCRA results indicated that BT + LI (97.6%) had the highest ranking probability, followed by LI (68.3%), Diet (56.6%), and Control (24.9%). WMSSP (2.6%) had the lowest ranking probability (**Figure 6**). However, the BDI-II network was highly sparse, with most interventions connected only through indirect comparisons, resulting in substantial uncertainty in SUCRA rankings. Therefore, these rankings should not be interpreted as evidence of true superiority.

#### 3.5.3 PHQ-9

CBT-CI notably reduced PHQ-9 scores compared to Diet (MD: -3.49, 95% CI: -5.13, -1.85), CBI (MD: -3.07, 95% CI: -4.66, -1.48), LI + MHS (MD: -2.6, 95% CI: -4.17, -1.03), and Control (MD: -3.33, 95% CI: -4.8, -1.86) (**Table 5**).

**Table 5:** League table for PHQ-9.

No.	X1	X2	X3	X4	X5	X6
1	1	-3.33 (-4.8, -1.86)	-1.69 (-3.77, 0.38)	-0.26 (-0.86, 0.34)	0.16 (-0.56, 0.88)	-0.73 (-1.29, -0.17)
2	3.33 (1.86, 4.8)	2	1.64 (-0.9, 4.18)	3.07 (1.48, 4.66)	3.49 (1.85, 5.13)	2.6 (1.03, 4.17)
3	1.69 (-0.38, 3.77)	-1.64 (-4.18, 0.9)	3	1.43 (-0.72, 3.61)	1.85 (-0.35, 4.05)	0.96 (-1.19, 3.12)
4	0.26 (-0.34, 0.86)	-3.07 (-4.66, -1.48)	-1.43 (-3.61, 0.72)	4	0.42 (-0.22, 1.05)	-0.47 (-1.29, 0.34)
5	-0.16 (-0.88, 0.56)	-3.49 (-5.13, -1.85)	-1.85 (-4.05, 0.35)	-0.42 (-1.05, 0.22)	5	-0.89 (-1.8, 0.02)
6	0.73 (0.17, 1.29)	-2.6 (-4.17, -1.03)	-0.96 (-3.12, 1.19)	0.47 (-0.34, 1.29)	0.89 (-0.02, 1.8)	6

**Figure 7:**
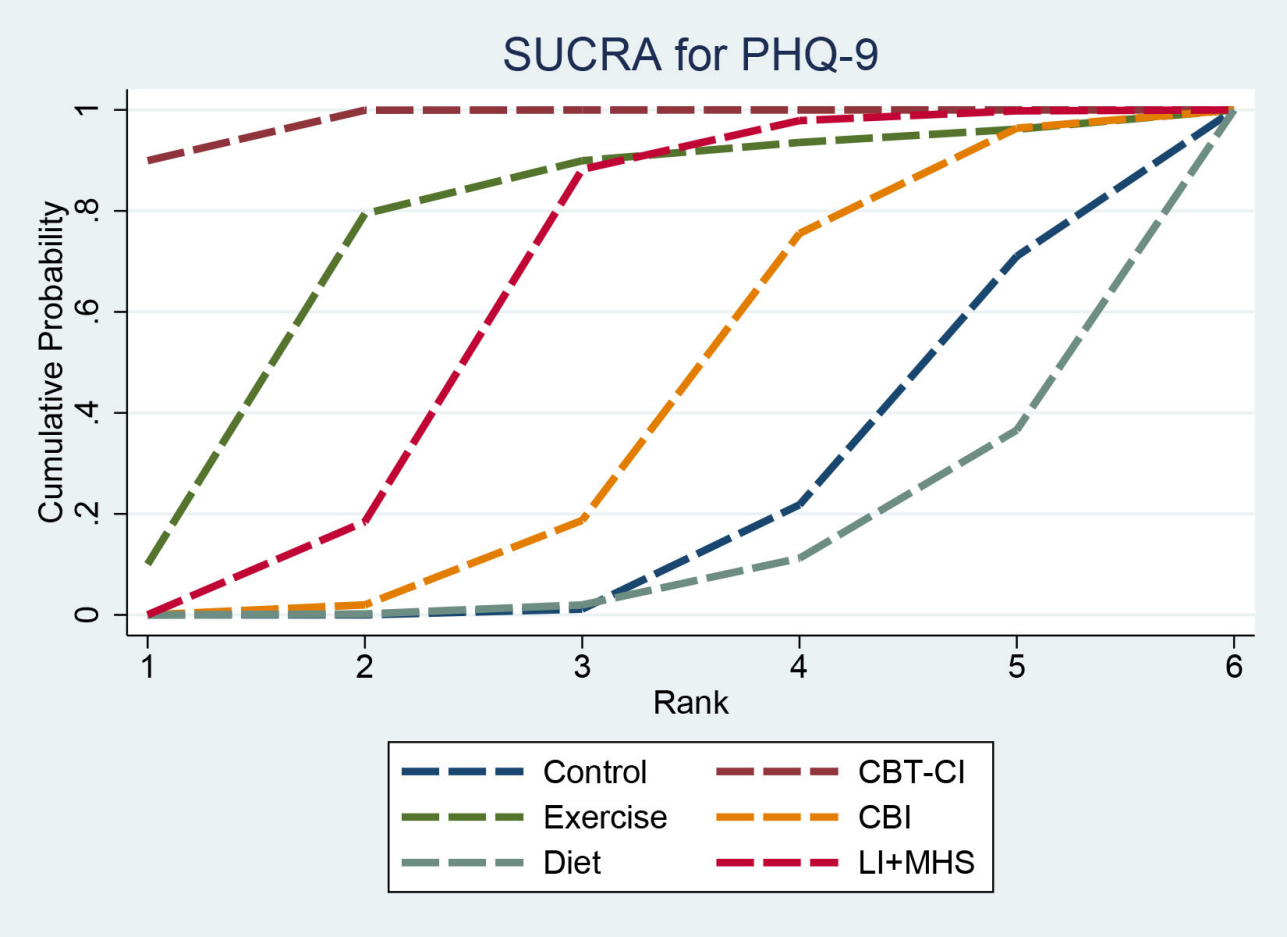
SUCRA for PHQ-9. SUCRA, surface under the cumulative ranking curve; PHQ-9, Patient Health Questionnaire-9.

SUCRA results indicated that CBT-CI (97.9%) had the highest ranking probability, followed by Exercise (74.2%), LI + MHS (60.6%), CBI (38.6%), and Control (18.5%), while Diet (10.2%) had the lowest ranking probability (**Figure 7**).

#### 3.5.4 CES-D

Compared to GM+TC, PMBI (MD: -9.34, 95% CI: -14.59, -4.1) significantly diminished depression scores, followed by DCI (MD: -9.11, 95% CI: -16.66, -1.56) and Diet (MD: -8.15, 95% CI: -12.61, -3.64) (**Table 6**).

**Table 6:** League table for CES-D.

No.	X1	X2	X3	X4	X5	X6	X7
1	1	-6.55 (-10.93, -2.13)	-7.51 (-14.98, -0.02)	-7.74 (-12.91, -2.6)	-1.2 (-3.72, 1.29)	-3.62 (-6.4, -0.85)	1.6 (0.67, 2.53)
2	6.55 (2.13, 10.93)	2	-0.97 (-7, 5.02)	-1.2 (-8.02, 5.57)	5.34 (0.21, 10.41)	2.93 (-2.3, 8.1)	8.15 (3.64, 12.61)
3	7.51 (0.02, 14.98)	0.97 (-5.02, 7)	3	-0.25 (-9.3, 8.88)	6.28 (-1.59, 14.2)	3.88 (-4.08, 11.86)	9.11 (1.56, 16.66)
4	7.74 (2.6, 12.91)	1.2 (-5.57, 8.02)	0.25 (-8.88, 9.3)	4	6.54 (2.02, 11.03)	4.13 (-1.73, 10.01)	9.34 (4.1, 14.59)
5	1.2 (-1.29, 3.72)	-5.34 (-10.41, -0.21)	-6.28 (-14.2, 1.59)	-6.54 (-11.03, -2.02)	5	-2.42 (-6.16, 1.33)	2.81 (0.13, 5.5)
6	3.62 (0.85, 6.4)	-2.93 (-8.1, 2.3)	-3.88 (-11.86, 4.08)	-4.13 (-10.01, 1.73)	2.42 (-1.33, 6.16)	6	5.21 (2.29, 8.15)
7	-1.6 (-2.53, -0.67)	-8.15 (-12.61, -3.64)	-9.11 (-16.66, -1.56)	-9.34 (-14.59, -4.1)	-2.81 (-5.5, -0.13)	-5.21 (-8.15, -2.29)	7

**Figure 8:**
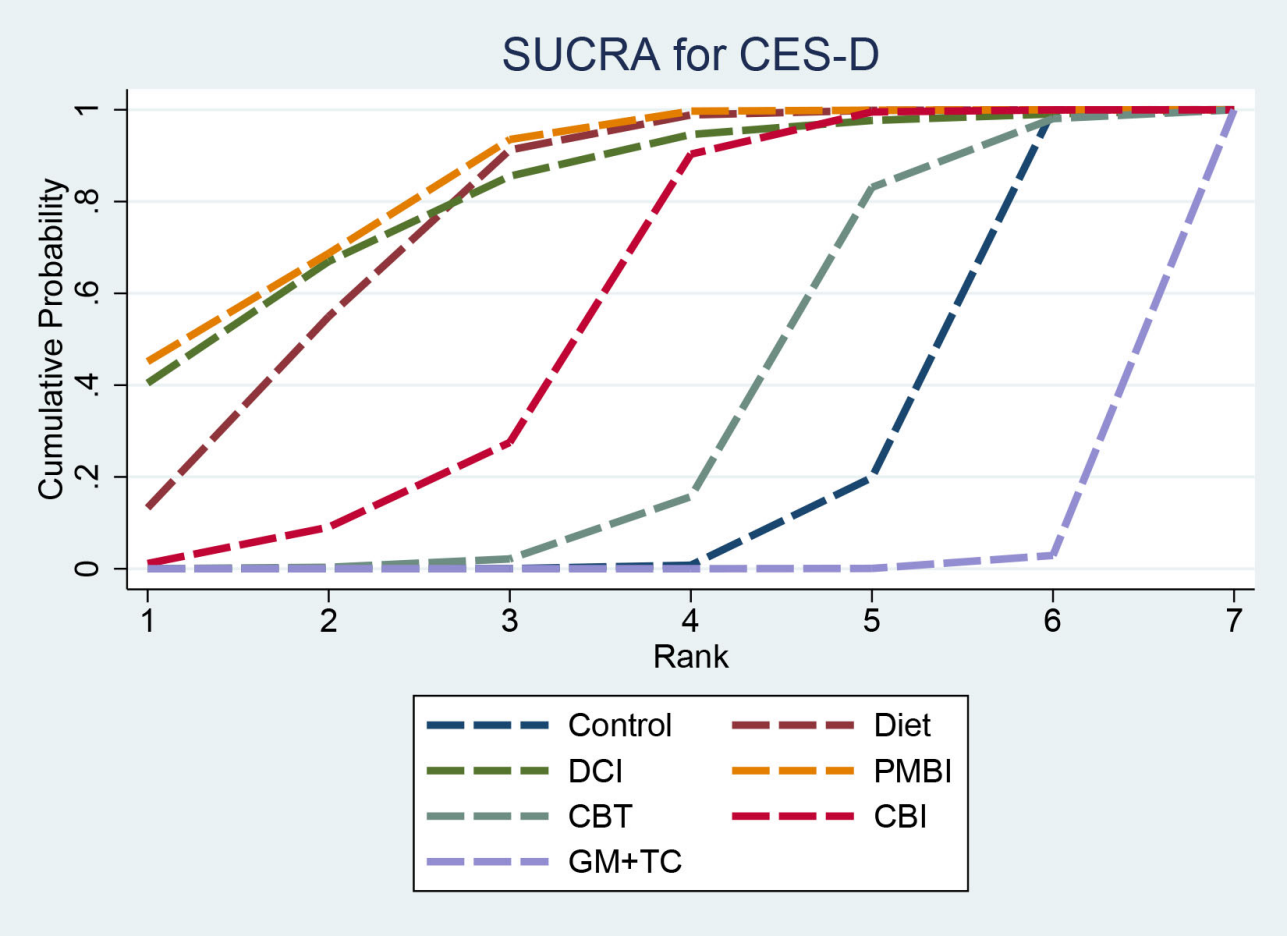
SUCRA for CES-D. SUCRA, surface under the cumulative ranking curve; CES-D, Center for Epidemiologic Studies Depression Scale.

SUCRA results indicated that PMBI (84.5%) had a higher ranking probability, followed by DCI (80.7%) and Diet (76.4%); GM+TC (0.5%) had the lowest ranking probability (**Figure 8**).

#### 3.5.5 HADS

League table results presented no statistical differences between intervention groups (**Table 7**).

**Table 7:** League table for HADS.

No.	X1	X2	X3	X4	X5
1	1	-4.23 (-10.21, 1.8)	-1.21 (-9.67, 7.35)	-1.2 (-7.01, 4.6)	-1.26 (-5.53, 2.77)
2	4.23 (-1.8, 10.21)	2	3.02 (-7.42, 13.5)	3.02 (-5.32, 11.44)	2.98 (-4.5, 10.17)
3	1.21 (-7.35, 9.67)	-3.02 (-13.5, 7.42)	3	0.01 (-6.32, 6.28)	-0.06 (-9.69, 9.22)
4	1.2 (-4.6, 7.01)	-3.02 (-11.44, 5.32)	-0.01 (-6.28, 6.32)	4	-0.05 (-7.35, 6.93)
5	1.26 (-2.77, 5.53)	-2.98 (-10.17, 4.5)	0.06 (-9.22, 9.69)	0.05 (-6.93, 7.35)	5

**Figure 9:**
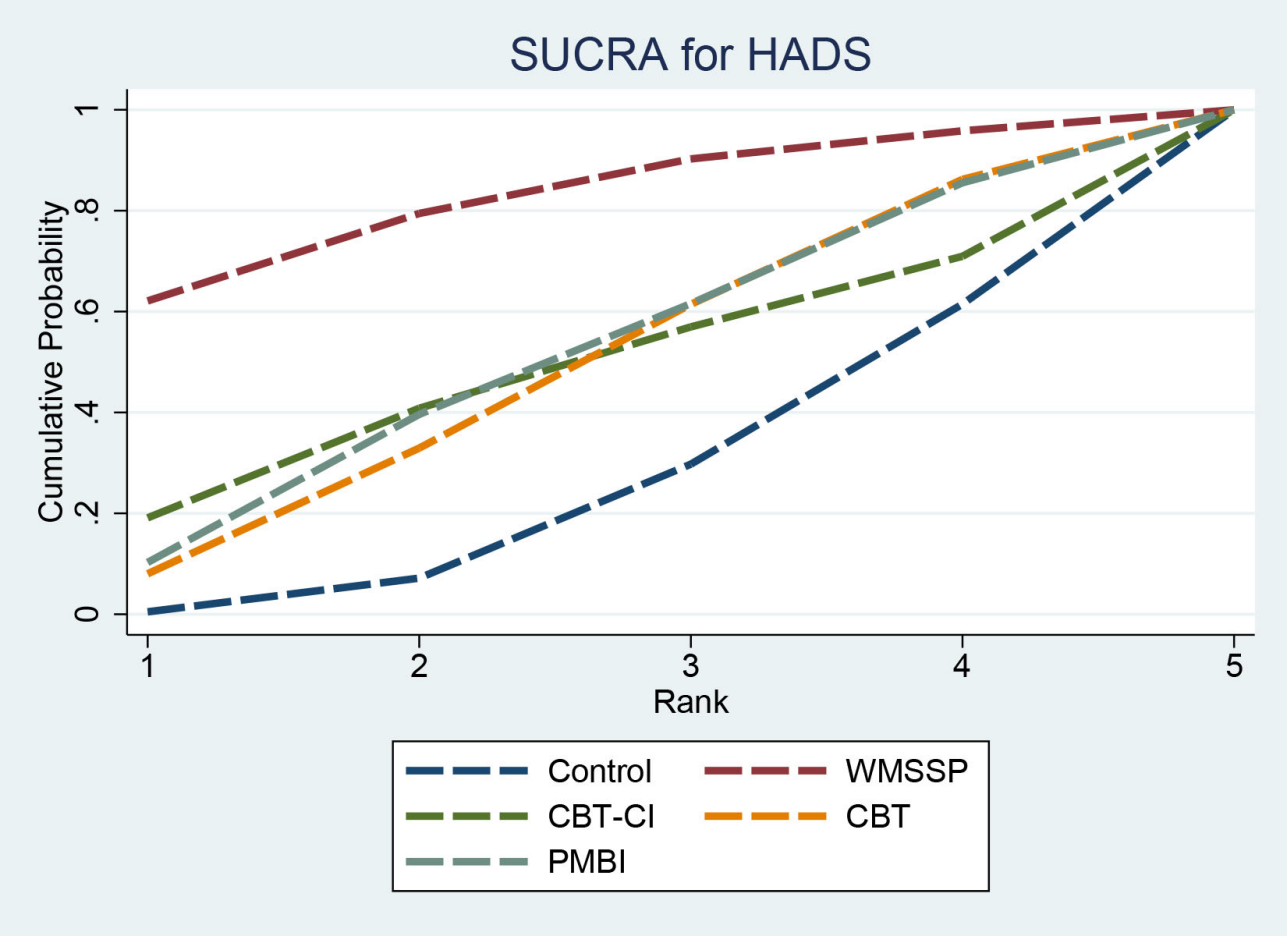
SUCRA for HADS. SUCRA, surface under the cumulative ranking curve; HADS, Hospital Anxiety and Depression Scale.

SUCRA results indicated that WMSSP (81.9%) had a higher ranking probability, followed by PMBI (49.3%), CBT (47.1%), and CBT-CI (47.0%), while Control (24.7%) had a lower ranking probability (**Figure 9**). Although WMSSP ranked highest in SUCRA, the HADS network was sparse and largely dependent on indirect comparisons, and the league table showed no statistically significant differences between interventions. Therefore, the ranking should be interpreted cautiously and does not indicate the true superiority of WMSSP.

To facilitate interpretation, a summary table of SUCRA values for all interventions across different depression scales is provided in **Tables 8**–**13**.

**Table 8:** SUCRA rankings for summary data of outcomes.

Id	Treatment	SUCRA results
1	Control	25.5%
2	CT	46.4%
3	BT	37.8%
4	CBI	61.5%
5	Pilates	37.5%
6	Exercise	38.0%
7	CBT	77.8%
8	CBT-CI	73.6%
9	Diet+Exercise	39.0%
10	WMSSP	47.3%
11	Diet	45.6%
12	LI	61.8%
13	BT+LI	74.0%
14	LI+MHS	41.9%
15	PMBI	67.8%
16	DCI	52.5%
17	GM+TC	23.7%

**Table 9:** SUCRA rankings for BDI.

Id	Treatment	SUCRA results
1	Control	26.4%
2	CT	42.2%
3	BT	34.4%
4	CBI	66.9%
5	Pilates	36.6%
6	Exercise	40.1%
7	CBT	94.9%
8	CBT-CI	85.5%
9	Diet+Exercise	34.8%
10	WMSSP	52.7%
11	Diet	35.5%

**Table 10:** SUCRA rankings for BDI-II.

Id	Treatment	SUCRA results
1	Control	24.9%
2	LI	68.3%
3	BT+LI	97.6%
4	WMSSP	2.6%
5	Diet	56.6%

**Table 11:** SUCRA rankings for PHQ-9.

Id	Treatment	SUCRA results
1	Control	18.5%
2	CBT-CI	97.9%
3	Exercise	74.2%
4	CBI	38.6%
5	Diet	10.2%
6	LI+MHS	60.6%

**Table 12:** SUCRA rankings for CES-D.

Id	Treatment	SUCRA results
1	Control	20.1%
2	Diet	76.4%
3	DCI	80.7%
4	PMBI	84.5%
5	CBT	33.2%
6	CBI	54.6%
7	GM+TC	0.5%

**Table 13:** SUCRA rankings for HADS.

Id	Treatment	SUCRA results
1	Control	24.7%
2	WMSSP	81.9%
3	CBT-CI	47.0%
4	CBT	47.1%
5	PMBI	49.3%

### 3.6 Publication bias

**Figure 10:**
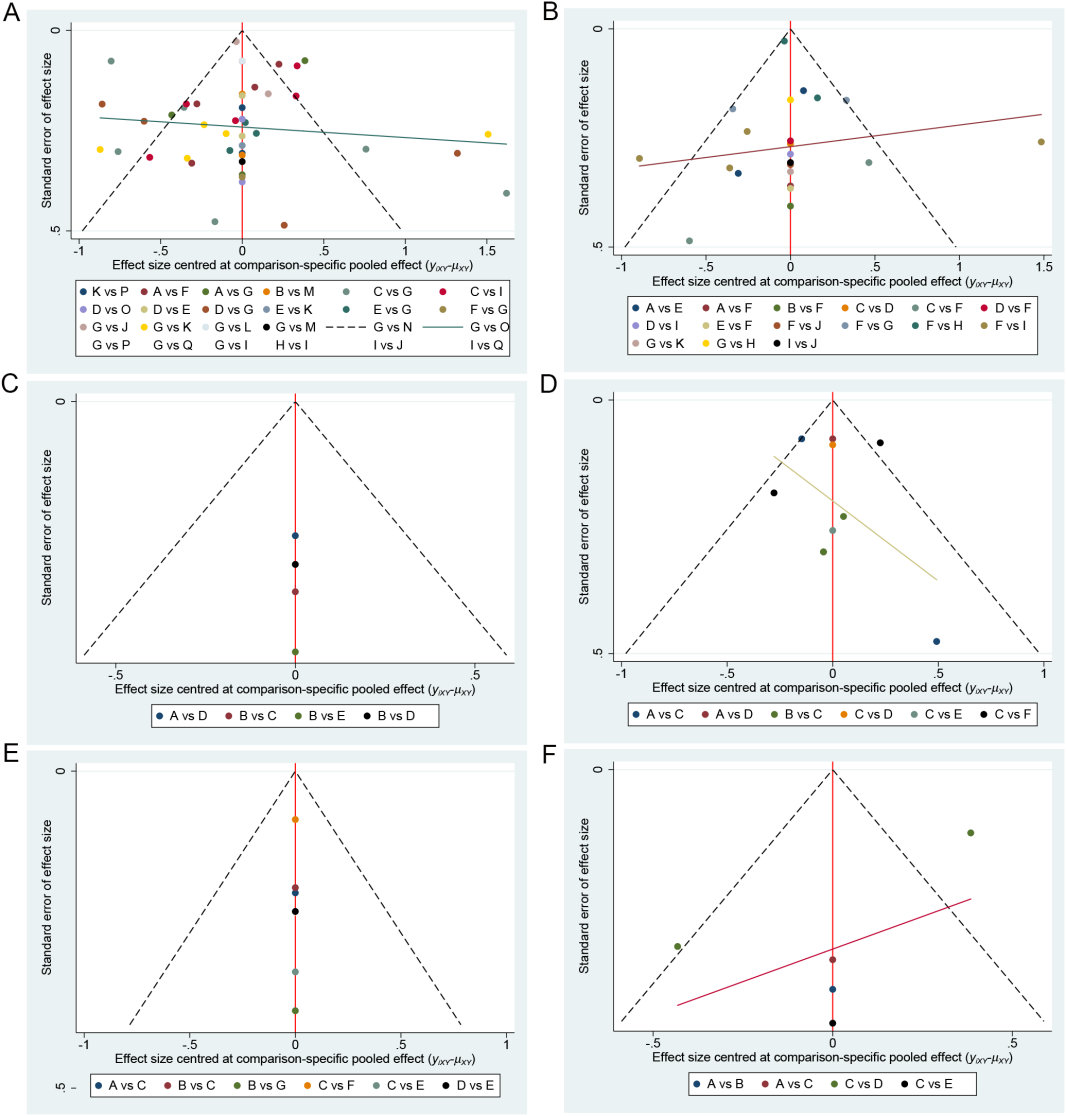
Funnel plot. **(A)** Funnel Plot for Summary Data; **(B)** BDI Funnel Plot; **(C)** BDI-II Funnel Plot; **(D)** PHQ-9 Funnel Plot; **(E)** CES-D Funnel Plot; **(F)** HADS Funnel Plot. BDI, Beck Depression Inventory; PHQ-9, Patient Health Questionnaire-9; HADS, Hospital Anxiety and Depression Scale.

The funnel plot demonstrated the presence of publication bias in the summary data. Among the five assessment scales, the BDI and HADS showed potential publication bias. The remaining scales generally exhibited symmetry between the left and right sides of the funnel plot, suggesting lower levels of publication bias (**Figures 10A–F**).

### 3.7 Heterogeneity and inconsistency tests

Heterogeneity analysis revealed pronounced heterogeneity in CBI, Exercise, CBT, WMSSP, Diet, LI+MHS, and PMBI compared to Control based on summary data. Additionally, high heterogeneity existed between CBT and PMBI, as well as between CBT-CI and Exercise. Studies using BDI as the outcome measure showed greater heterogeneity in Exercise and CBT-CI compared to Control. In studies using the PHQ-9 as an outcome measure, CBI and Diet demonstrated higher heterogeneity compared to Control; in studies using the HADS as an outcome measure, PMBI showed higher heterogeneity compared to Control. The observed heterogeneity may stem from differences in intervention intensity, frequency, baseline characteristics of the study populations, and study design. For the remaining interventions under the depression scales, the I² values were all below 50% or not comparable, indicating low heterogeneity (**Appendix 2**). Additionally, while the loop-specific τ² values for composite data, BDI, and PHQ-9 suggested heterogeneity within individual triangular loops, these values were generally small and exhibited limited distribution. Consequently, their impact on main effect estimates and SUCRA rankings is likely minimal. Meanwhile, since BDI-II, CES-D, and HADS did not form an evaluable triangular or quadrilateral loop, their τ² could not be estimated. This structural limitation is also unlikely to substantially undermine the stability of the overall conclusions (**Appendix 3**). The inconsistency test employed the node splitting method. Among the summary data, the CBT vs Control comparison showed inconsistency (p = 0.040), and statistically significant inconsistency was identified between PMBI and the control group in the summary data (p = 7.5×10^-5^), as well as between PMBI and CBT (p = 5×10^-5^), while all other results were consistent (p ≥ 0.05). This divergence may stem from heterogeneity in intervention details or baseline characteristics of study subjects across studies providing direct versus indirect evidence, necessitating cautious interpretation, particularly for PMBI-related comparisons. The comparison of CBT-CI vs Exercise in the BDI subgroup showed marginal inconsistency (p = 0.032), suggesting potential conflict between direct and indirect evidence at this specific comparison node. This inconsistency was confined to a single local comparison, while the global model demonstrated adequate overall consistency, supporting the robustness of the SUCRA rankings and main comparisons (**Appendix 4**).

### 3.8 Sensitivity analysis

To assess the robustness of the NMA results, sensitivity analyses were conducted on the summary data and CES-D scale data. This was due to one included study featuring adolescent rather than adult participants (16). The results indicated that the primary conclusions remained generally robust after excluding this study. In the sensitivity analysis of summary data, compared with the primary analysis results, the effect sizes and their 95% CIs for each intervention versus the control group showed no shift in direction, and the conclusions regarding statistical significance remained consistent. CBT continued to demonstrate the most favorable reduction effect (SMD: -6.94, 95% CI: -11.17 to -2.80). The SUCRA ranking order of other interventions also remained largely unchanged, indicating robust analytical results from the summary data.

In the sensitivity analysis of the CES-D scale, we removed PMBI data to assess its impact. Results indicated that comparisons among the remaining interventions remained stable. The efficacy advantage of DCI and Diet over GM+TC remained significant. The SUCRA ranking order of primary interventions showed no fundamental change compared to the analysis including PMBI, demonstrating the robustness of comparison results across the rest of the CES-D network beyond the PMBI node. This finding suggested that PMBI’s superior performance was a significant and independent driver of the primary CES-D analysis results (**Appendix 5**).

## 4 Discussion

This NMA represents the first systematic attempt to integrate 16 non-pharmacological interventions to evaluate their efficacy in individuals with obesity and comorbid depression, filling an evidence gap in comparative non-pharmacological interventions in this field (10). A multidimensional efficacy ranking was constructed based on five depression scales, and SUCRA analysis was employed to quantify intervention priority, providing evidence-based guidance for clinical practice.

NMA analysis revealed that CBT and its derivative forms (e.g., CBT-CI) demonstrated optimal improvements in depression across comprehensive data analysis. In scale-specific analyses, CBT-based interventions showed meaningful effects on BDI and PHQ-9 scales, PMBI worked relatively well in CES-D assessments, and BT+LI showed meaningful efficacy in the BDI-II assessment. Overall, CBT-based interventions appeared relatively strong in relieving depressive symptoms among obese participants, while PMBI and BT+LI showed relatively strong outcomes in combined interventions. Notably, comparisons among interventions using HADS as an assessment tool yielded no statistical differences, potentially attributable to scale characteristics and sample composition.

The findings are largely consistent with previous meta-analyses and research reports, though minor discrepancies exist. CBT has been widely applied across diverse populations for its proven efficacy in relieving depressive symptoms and preventing relapse (21–23). In patients with comorbid obesity and depression, it achieves dual goals of weight loss and mood improvement (24, 25) but also enables continuous service delivery during exceptional periods like the COVID-19 pandemic through integration with information technology (e.g., Tele-CBT) (26, 27). Multiple studies indicate that BT and LI can substantially improve depressive symptoms in overweight and obese individuals. A study published in the International Journal of Obesity demonstrated that BT+LI effectively palliated depressive symptoms in overweight/obese populations (28); Another systematic review and meta-analysis found that LI can help overweight and obese individuals with severe mental illness achieve weight loss, thereby indirectly improving their mental health (29). PMBI shows potential in improving multiple health conditions. Meta-analyses indicate that general psychosocial interventions can effectively relieve depressive symptoms in study participants (30). Weiyu Mao et al. also suggested that MBI and other psychosomatic interventions could effectively reduce depressive symptoms in older adults within a short period (31). Research also found that adolescents receiving PMBI demonstrated improvements in both depressive symptoms and metabolic indicators during long-term follow-up, suggesting this intervention may offer sustained benefits (16). Notably, single-dimensional interventions often yield limited effects. For instance, this NMA found that BT alone did not demonstrate significant efficacy. This may stem from its narrow focus on behavioral modification, neglecting multidimensional factors such as cognitive restructuring, emotional regulation, or social support. Consequently, it struggles to comprehensively address the complex psychophysiological conditions of obese individuals with depression.

CBT and its derivative approaches can substantially alleviate depressive symptoms, primarily due to their distinctive intervention paradigms. These therapies not only assist patients in identifying and modifying negative thought patterns but also effectively manage emotional eating and exercise avoidance behaviors (32). The core of CBT lies in identifying and challenging patients’ negative and unrealistic thoughts (33). For obese individuals, these thoughts may include negative self-evaluations of body weight, pessimistic expectations about weight loss, and doubts about personal capabilities. These perceptions generate anxiety, low self-esteem, and shame, which in turn exacerbate depressive symptoms (34). CBT employs cognitive restructuring techniques to help patients recognize these thoughts as inaccurate and replace them with more positive, realistic ones (35), while teaching patients skills to identify and manage these emotions (36). For example, mindfulness exercises enhance emotional awareness, while cognitive techniques modify responses to emotions (37). Additionally, CBT emphasizes the role of behavioral change in alleviating depressive symptoms. For obese individuals, this entails modifying unhealthy dietary and exercise habits to cultivate a healthier lifestyle (38). Through these behavioral modifications, patients can not only achieve weight loss but also enhance self-esteem and self-efficacy, thereby lessening depressive symptoms (39). Derivative interventions of CBT, particularly remote delivery methods, such as Tele-CBT and IT + CBT, considerably enhance treatment accessibility and continuity while preserving the core strengths of traditional CBT. This enables more patients to receive timely psychological support (27, 40, 41).

BT+LI also demonstrated significant efficacy, with its mechanism of action rooted in integrating behavioral change with lifestyle adjustments. BT aims to help patients identify and engage in activities that bring pleasure and a sense of accomplishment, encouraging proactive action even during depressed moods to activate positive feedback loops (42). LI emphasizes achieving health goals by modifying daily habits and behavioral patterns (43, 44). When combined, these approaches effectively improve patients’ exercise adherence and dietary behaviors by setting specific behavioral goals, establishing self-monitoring mechanisms, and providing social support (45).

PMBI incorporates psychosocial interventions and mindfulness interventions. As a multidimensional integrated intervention strategy, its efficacy stems from a mechanism involving synergistic effects across multiple pathways (46). Through core components like mindfulness training, emotional awareness, and acceptance, PMBI enhances individuals’ capacity to regulate stress and negative emotions, reduces stress-related behaviors (emotional eating), and consequently improves metabolic indicators and psychological well-being (47, 48). Such interventions typically incorporate elements of group support and social connection. By promoting interpersonal learning, reducing illness-related stigma, and enhancing a sense of belonging, they alleviate social isolation and self-criticism commonly experienced by obese individuals, thereby mitigating depressive symptoms (49). Moreover, PMBI emphasizes mind-body interaction. Through somatic practices (yoga and breathing exercises), it regulates autonomic nervous system function and reduces cortisol levels, thereby improving insulin resistance and inflammatory states caused by chronic stress (50, 51). This approach further eliminates triggers of depressive symptoms, thereby indirectly promoting emotional regulation (52). Unlike traditional behavioral interventions that primarily focus on external goals, such as weight numbers and calorie control, the core mechanism of PMBI lies in enhancing psychological flexibility and reducing avoidance of negative experiences through mindfulness and acceptance strategies. It also alleviates self-critical thinking commonly found in obese individuals through self-compassion training. This approach breaks the vicious cycle of stress and emotional eating, thereby mitigating depressive symptoms at their root and promoting sustainable healthy behaviors (53). However, the specific pathways of action and optimal populations for PMBI require further validation through high-quality research.

Based on the pooled evidence and efficacy rankings, we recommend CBT and its related derivative interventions as the preferred treatment option, particularly for patient groups requiring simultaneous improvements in emotional and cognitive functioning. For patients with significant lifestyle risk factors, comprehensive interventions, such as BT+LI or PMBI, are more suitable. In clinical practice, individualized treatment plans should be developed based on the patient’s symptom profile, response patterns to assessment tools, and personal acceptance.

This study has several limitations (1). The included populations exhibited significant heterogeneity, limiting the generalizability of findings. The wide age range of participants—spanning multiple developmental stages from adolescence to old age—may reflect fundamental differences in depressive mechanisms and intervention responses. To further assess the transitivity assumption in our NMA, we compared key clinical characteristics across interventions, including age, baseline depression severity, use of antidepressants, and delivery format (**Table 14**). In addition, as with all network meta-analyses, the validity of indirect comparisons relies on the assumption of transitivity, which may be affected by variability across populations, interventions, and outcome measures. Furthermore, the highly skewed sex distribution, coupled with the absence of sex-specific subgroup analyses, precludes assessment of potential sex-specific effects of interventions, introducing potential bias. In addition, the geographic distribution of the included studies may limit external validity. All eligible trials were conducted in high- or very-high–HDI countries, predominantly in Western regions, with no studies from Africa and only limited studies from Asia and Latin America. Given the cultural sensitivity of non-pharmacological, particularly psychological, interventions, the applicability of these findings to lower-HDI or socioeconomically disadvantaged settings remains uncertain. This underrepresentation may partly reflect structural and resource constraints in less-developed regions, where conducting large-scale randomized controlled trials can be challenging due to limited research infrastructure and funding. Future studies in diverse socioeconomic and cultural contexts are therefore warranted to enhance the global applicability of non-pharmacological interventions. This limitation is partly attributable to the databases and language restrictions applied, as region-specific databases were not searched and non-English publications were excluded, potentially introducing geographic and language bias (2). The lack of uniform standards across intervention types compromises the accuracy of efficacy comparisons. Although studies covered multiple intervention modalities, significant variations existed in the implementation details of similar interventions. For instance, specific interaction frequencies in CBT-CI, exercise intensity and frequency in exercise interventions, calorie restriction levels in LI, and behavioral support durations remain unstandardized. Such operational inconsistencies make it difficult for NMAs to distinguish between the effects of the interventions themselves and the impact of implementation variations on outcomes. This inconsistency may particularly undermine the reliability of ranking results for complex interventions like CBT and LI (3). Statistically significant inconsistencies were detected in the node-splitting analysis, specifically in the comparisons of PMBI versus the control group (p = 7.5×10^-5^) and PMBI versus CBT (p = 5×10^-5^). These inconsistencies may reflect limited direct evidence, variations in intervention delivery, or differences in baseline depression severity among PMBI-related trials. Although these isolated inconsistencies did not materially affect the SUCRA rankings or the overall conclusions of the NMA, they warrant cautious interpretation (4). Insufficient evidence on long-term efficacy limits the guidance value of findings for chronic disease management. Most studies had short intervention periods, with only a few trials reaching follow-up durations of one year or longer. Both obesity and depression are highly recurrent conditions, and short-term improvements do not predict long-term outcomes. The current SUCRA ranking primarily reflects short-term effects, failing to address key questions such as the preventive effect of CBT on depression recurrence or the long-term maintenance of weight through lifestyle changes (5). Differences in dimensional characteristics among various depression assessment tools may influence the presentation of intervention effects. The HADS scale contains many anxiety items, making its scores susceptible to interference from comorbid anxiety symptoms; the CES-D scale focuses on somatic symptoms such as sleep and appetite, rendering it more responsive to physiologically oriented interventions like dietary and exercise programs. Although this NMA employed SMD and subscale-based evidence networks to mitigate heterogeneity, differences in psychometric properties between scales are difficult to fully eliminate. This may partly explain why CBT demonstrated superior performance on cognitive-emotional scales like the BDI, while Diet showed greater efficacy on scales emphasizing somatic symptoms, such as the CES-D. This phenomenon does not negate the validity of cross-scale comparisons but rather suggests that clinical interpretations should be made holistically by considering the structural characteristics of specific assessment tools and their intervention mechanisms (6). Evidence networks in certain areas are relatively weak, reducing estimation accuracy. Comparisons between some interventions rely on a single study. For instance, the BDI-II network lacked direct comparative evidence for certain nodes, and the transmissibility assumptions underlying indirect comparisons may not be valid. The extremely low SUCRA scores for GM+TC across multiple networks are also linked to sparse direct evidence, necessitating cautious interpretation of its efficacy assessments. In addition, several interventions—particularly WMSSP and BT+LI—were connected to the evidence network primarily through indirect comparisons against the control group, with few or no closed loops formed to support consistency assessments. This sparse connectivity substantially increases the uncertainty of the corresponding effect estimates and limits the robustness of the SUCRA rankings. As a result, findings that rely predominantly on indirect evidence should be interpreted with caution (7). The inconsistent methodological quality of original RCTs may also confound the results. The ROB 2.0 assessment indicated that some RCTs carried a risk of bias in random sequence generation, allocation concealment, or blinding of outcomes. Although this NMA employed a random-effects model and sensitivity analyses to mitigate these risks, it could not eliminate the impact of such biases on the pooled results (8). Another limitation is the presence of local inconsistency in CBT-related comparisons. This inconsistency may reflect variations in CBT delivery formats or intervention components across studies, which could affect the stability of pooled estimates. Accordingly, CBT-related findings should be interpreted with caution.

**Table 14:** Summary of key clinical and methodological characteristics across included trials.

Id	Author	Year	Country	Treatment	Delivery format	Sample	Age	Baseline depression severity	Use of antidepressants
1	Tanco	1998	Canada	Cognitive therapy(CT)	Group-based	18	NR	17.9 ± 10.5	NR
				Behavioral therapy(BT)	Group-based	19	NR	15.8 ± 8.4	NR
				Control	Control	13	NR	19.6 ± 7.8	NR
2	Freitas	2018	Brazil	Weight Management and Structured Support Programs(WMSSP)	Mixed/Hybrid	26	45.9 ± 7.7	9.6 ± 4.7	NR
				Control	Control	25	48.5 ± 9.6	9.6 ± 4.7	NR
3	Xenaki	2018	Brazil	Cognitive and Behavioral Interventions(CBI)	Mixed/Hybrid	22	46.9 ± 11.0	33.7 ± 4.9	Unused
				Control	Control	23	44.5 ± 10.1	29.6 ± 2.8	Unused
4	Werrij	2009	Netherlands	Cognitive therapy(CT)	Group-based	96	44.0 ± 11.9	10.0 ± 6.8	Unused
				Behavioral therapy(BT)	Group-based	104	45.0 ± 12.2	8.9 ± 7.1	Unused
5	Vancini	2017	Brazil	Control	Control	20	41.7 ± 12.6	11.9 ± 6.9	Unused
				Aerobic exercise	Group-based	21	42.4 ± 7.0	18.7 ± 6.9	Unused
				Pilates	Group-based	22	55.9 ± 6.6	16.7 ± 6.8	Unused
6	Uemura	2019	Japan	Diet	Mixed/Hybrid	22	62.0 ± 8.7	17.6 ± 13.6	NR
				Control	Control	22	63.3 ± 9.1	11.6 ± 8.0	NR
7	Thomson	2010	Australia	Diet	Group-based	30	29.3 ± 0.7	18.2 ± 2.5	NR
				Dietary Combined Intervention(DCI)	Group-based	64	29.3 ± 0.7	15.9 ± 3.3	NR
8	Sockalingam	2022	Canada	Cognitive-Behavioral Therapy–Based Combined Intervention(CBT-CI)	Telephone-based	40	47.7 ± 9.4	5.5 ± 0.9	NR
				Control	Control	41	47.5 ± 9.4	7.2 ± 6.9	NR
9	Sockalingam	2015	Canada	Cognitive-Behavioral Therapy–Based Combined Intervention(CBT-CI)	Telephone-based	23	45.5 ± 8.9	5.0 ± 4.5	NR
				Control	Control	24	45.5 ± 8.9	5.2 ± 4.8	NR
10	Shomaker	2019	America	Psychosocial and Mind–Body Interventions(PMBI)	Group-based	17	15.0 ± 1.7	25.3 ± 6.6	NR
				Cognitive behavioral therapy(CBT)	Group-based	15	15.0 ± 1.7	25.3 ± 6.6	NR
11	Shomaker	2016	America	Cognitive behavioral therapy(CBT)	Group-based	61	NR	NR	Unused
				Control	Control	58	NR	NR	Unused
12	Shelley	2007	Canada	Cognitive behavioral therapy(CBT)	Group-based	13	NR	19.7 ± 9.7	Unused
				Control	Control	9	NR	16.4 ± 8.6	Unused
13	Seo	2023	South Korea	Exercise	Individual, home-based	47	48.4 ± 6.2	5.7 ± 3.9	NR
				Control	Control	23	48.3 ± 7.6	6.0 ± 2.8	NR
14	Sarsan	2006	Türkiye	Exercise	Group-based	40	42.5 ± 8.9	13.1 ± 6.28	Unused
				Control	Control	20	43.6 ± 6.5	14.85 ± 9.25	Unused
15	Painot	2001	Netherlands	Cognitive-Behavioral Therapy–Based Combined Intervention(CBT-CI)	Group-based	25	42 ± 2.0	17.0 ± 2.0	Unused
				Cognitive behavioral therapy(CBT)	Group-based	35	44 ± 2.0	17.0 ± 2.0	Unused
16	Pagoto	2013	America	Behavioral therapy+Lifestyle intervention(BT+LI)	Mixed/Hybrid	78	45.6 ± 11.0	21.1 ± 5.7	Use of antidepressants:29.5%
				Control	Control	83	46.2 ± 10.8	21.0 ± 5.9	Use of antidepressants:31.1%
17	Owens	2021	Netherlands, Spain, Germany, Britain	Cognitive and Behavioral Interventions(CBI)	Mixed/Hybrid	513	47.5 ± 13.0	7.2 ± 4.2	Unused
				Control	Control	256	47.5 ± 13.0	7.34 ± 4.14	Unused
				Diet	Mixed/Hybrid	256	47.5 ± 13.0	7.93 ± 4.39	Unused
18	Berman	2015	America	Cognitive and Behavioral Interventions(CBI)	Group-based	9	52.7 ± 8.4	14.9 ± 2.0	NR
				Control	Control	10	50.3 ± 17.4	12 ± 2.0	NR
19	Naparstek	2017	America	Cognitive and Behavioral Interventions(CBI)	Online/Web-based	83	46.4 ± 12.0	10.9 ± 7.4	NR
				Control	Control	42	47.8 ± 10.5	10.3 ± 7.2	NR
20	Moraes	2021	Brazil	Cognitive-Behavioral Therapy–Based Combined Intervention(CBT-CI)	Mixed/Hybrid	31	36.0 ± 6.8	16.94 ± 8.04	NR
				Exercise	Group-based	34	38.0 ± 6.0	15.17 ± 9.26	NR
				Control	Control	33	36.2 ± 2.8	16.00 ± 6.05	NR
21	Moncrieft	2016	America	Lifestyle intervention(LI)	Mixed/Hybrid	57	54.8 ± 8.3	19.3 ± 7.1	Use of antidepressants:14.04%
				Control	Control	54	54.8 ± 6.3	21.2 ± 7.1	Use of antidepressants:18.52%
22	Levine	1996	America	Exercise	Individual, home-based	44	36.3 ± 6.8	18.3 ± 7.8	NR
				Control	Control	33	37.0 ± 6.1	20.2 ± 7.8	NR
23	Holland-Carter	2017	America	Lifestyle intervention + Mental health support(LI+MHS)	Individual, face-to-face	279	55.2 ± 8.9	3.5 ± 3.6	NR
				Control	Control	284	54.9 ± 9.3	2.9 ± 3.2	NR
24	Heath	2022	Britain	Psychosocial and Mind–Body Interventions(PMBI)	Group-based	1056	53.5 ± 13.7	5.3 ± 3.5	NR
				Control	Control	211	51.9 ± 14.1	5.6 ± 3.8	NR
25	Gade	2015	Norway	Cognitive behavioral therapy(CBT)	Individual, face-to-face	42	44.1 ± 9.8	5.3 ± 3.8	NR
				Control	Control	38	41.2 ± 9.6	4.2 ± 2.8	NR
26	Faulconbridge	2012	America	Diet+Exercise	Mixed/Hybrid	2563	NR	5.5 ± 5.2	NR
				Control	Control	2566	NR	5.4 ± 4.7	NR
27	Drew	2022	Australia	Lifestyle intervention + Mental health support(LI+MHS)	Online/Web-based	62	48.4 ± 11.7	9.1 ± 4.2	NR
				Control	Control	63	48.4 ± 11.7	9.4 ± 4.0	NR
28	Demark-Wahnefried	2015	America	Group meetings + telephone consultations(GM+TC)	Mixed/Hybrid	344	56.0 ± 9.47	9.9 ± 0.5	NR
				Control	Control	348	56.4 ± 9.53	9.7 ± 0.5	NR
29	Cassin	2016	Canada	Cognitive-Behavioral Therapy–Based Combined Intervention(CBT-CI)	Telephone-based	23	45.5 ± 8.9	5.0 ± 4.5	NR
				Control	Control	24	45.5 ± 8.9	5.2 ± 4.8	NR
30	Bacon	2005	America	Weight Management and Structured Support Programs(WMSSP)	Group-based	19	41.4 ± 3.0	10.3 ± 9.5	NR
				Diet	Group-based	19	40.0 ± 4.4	7.5 ± 7.2	NR
31	Altazan	2019	America	Weight Management and Structured Support Programs(WMSSP)	Mixed/Hybrid	37	29.1 ± 4.4	6.6 ± 4.8	NR
				Control	Control	17	29.5 ± 5.1	8.8 ± 5.6	NR
32	Alfonsson	2015	Sweden	Psychosocial and Mind–Body Interventions(PMBI)	Group-based	50	45.5 ± 10.7	8.67 ± 4.50	Use of antidepressants:40.8%
				Control	Control	46	44.2 ± 10.9	7.72 ± 4.91	Use of antidepressants:31.8%
33	Abdollahi	2018	Iran	Cognitive behavioral therapy(CBT)	Group-based	37	28.4 ± 4.2	16.5 ± 6.0	Unused
				Control	Control	37	27.4 ± 4.6	13.0 ± 5.7	Unused
34	Lin	2023	America	Diet	Online/Web-based	60	44.0 ± 11.0	13.5 ± 10.2	NR
				Control	Control	30	44.0 ± 13.0	10 ± 8	NR
35	Kiernan	2001	America	Diet + Exercise	Group-based	39	38.5 ± 6.4	5.8 ± 4.9	NR
				Diet	Group-based	40	38.5 ± 6.4	5.4 ± 3.9	NR
				Control	Control	40	38.5 ± 6.4	5.9 ± 5.6	NR
36	Sanchez	2017	Canada	Diet	Individual, face-to-face	62	35.0 ± 10.0	4.4 ± 4.1	NR
				Control	Control	63	37.0 ± 10.0	4.7 ± 4.2	NR

NR, not reported.

Future research should focus on advancing non-pharmacological interventions toward standardization, individualization, and long-term sustainability. Priority should be given to establishing core elements and implementation standards for interventions targeting individuals with obesity and depression, particularly by specifying operational protocols for multi-component interventions such as CBT and PMBI. More high-quality, long-term RCTs are needed, with a focus on depression relapse prevention, weight maintenance, and sustainability of healthy behaviors. Additionally, responder models based on demographic characteristics, clinical phenotypes, and psychometric features should be explored to establish stratified intervention strategies. Cross-scale efficacy comparisons require integration of multidimensional indicators and individualized goals. Subsequent studies are advised to incorporate the dimensional characteristics of specific assessment tools and intervention mechanisms for clinical interpretation, thereby providing a more comprehensive and robust evidence base for clinical decision-making.

## 5 Conclusions

This NMA indicates that CBT and its derivative forms demonstrate relatively stronger overall effects in alleviating depressive symptoms among obese participants. However, considering key aspects of the GRADE principles, the certainty of evidence for most comparisons should be regarded as low or very low. PMBI shows relatively favorable performance in CES-D assessments, while BT+LI shows favorable results in the BDI-II scale. Although CBT-based interventions ranked highly in SUCRA, these findings should be interpreted cautiously because many included RCTs had some concerns or a high risk of bias. Additionally, given the inconsistency, imprecision, and reliance on indirect comparisons, the strength of inference is limited. Thus, CBT may be viewed as a potentially promising option rather than a definitive first-line treatment. Accordingly, the present findings should be interpreted as exploratory and hypothesis-generating rather than definitive conclusions for clinical recommendations. Rather than as a definitive treatment option, PMBI needs to be further investigated for patients with markedly elevated CES-D scores or those preferring mind-body integrated interventions. Those with prominent BDI-II scores may represent a population of interest for the BT+LI approach. Future high-quality randomized controlled trials with better intervention standardization, longer follow-up periods, and more diverse populations are needed to confirm these findings. Future research should also continue to focus on the long-term effects and specific mechanisms of different interventions.

## Data Availability

The original contributions presented in the study are included in the article/**Supplementary Material**. Further inquiries can be directed to the corresponding authors.
