# Supplementary figures and images for: Effect of non-pharmacological interventions on depression in obese individuals: a network meta-analysis
Source: Front Psychiatry. 2026 Feb 16;17:1715475. doi: 10.3389/fpsyt.2026.1715475 (PMC12950660; doi:10.3389/fpsyt.2026.1715475)

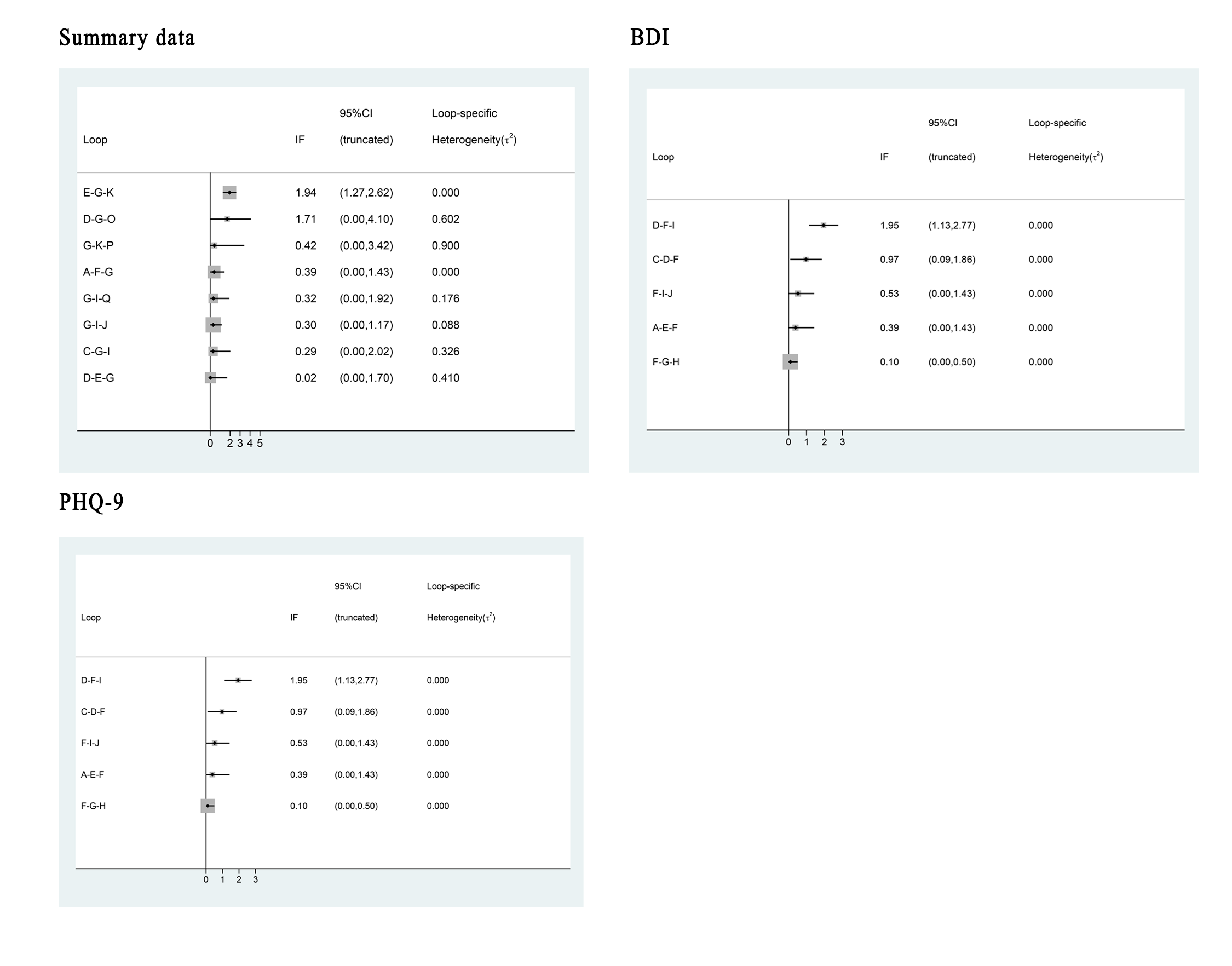

Supplement: Supplementary file 1 [file Image1.tif]

**Appendix 2** Heterogeneity Test

**BDI**


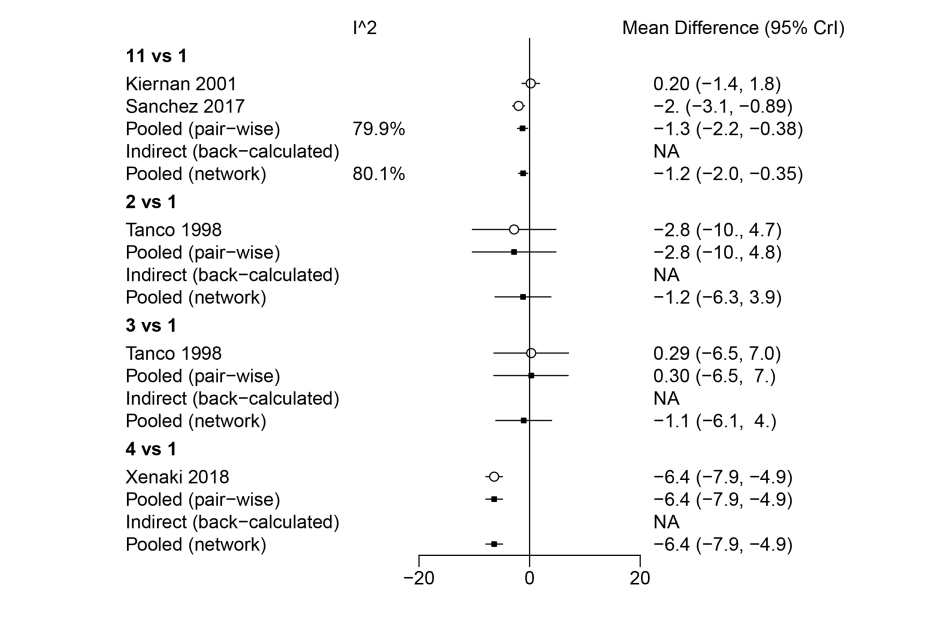

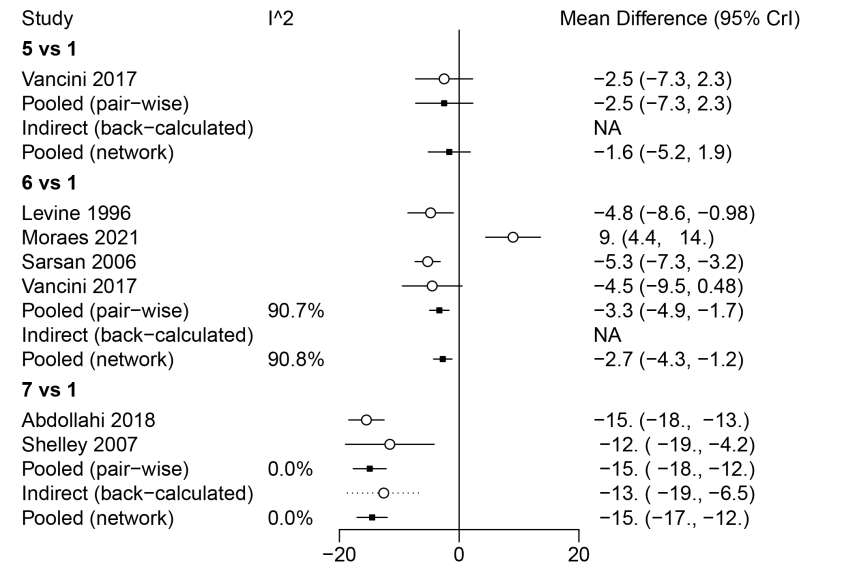


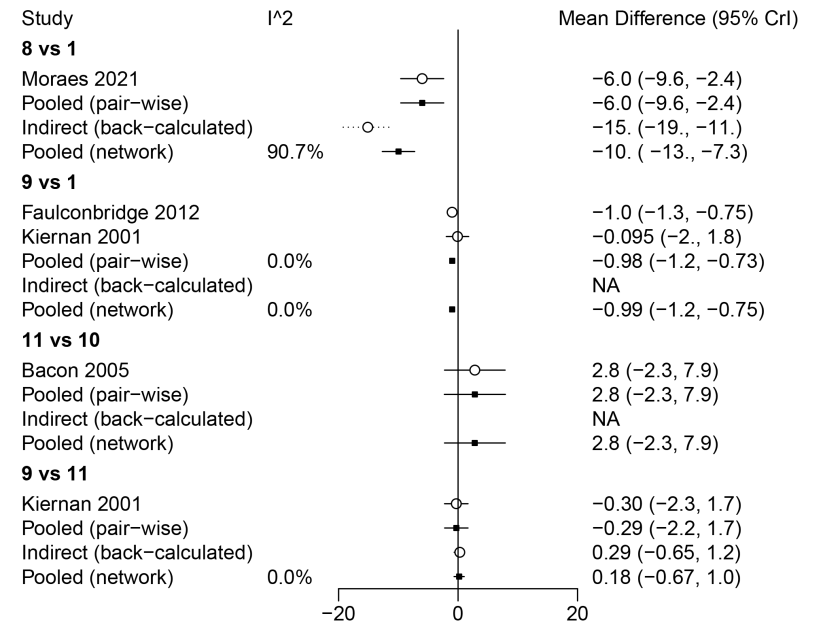

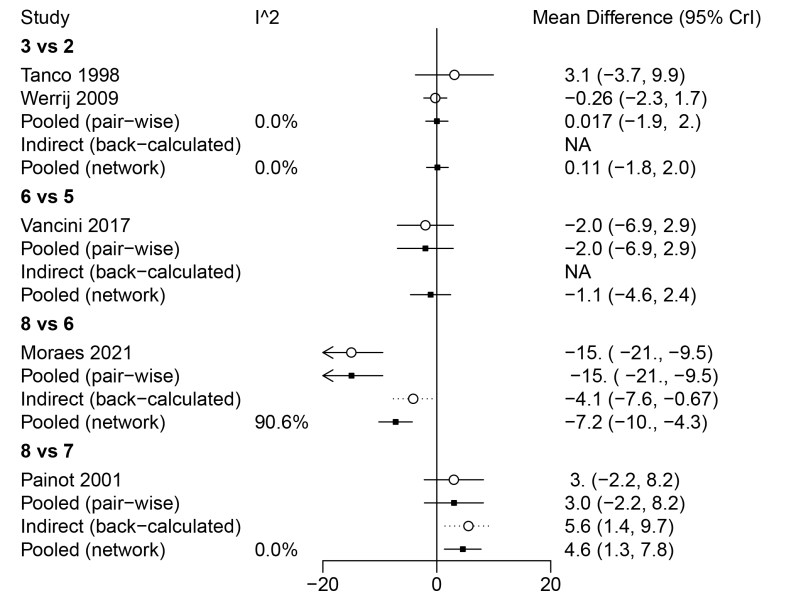


**HADS**


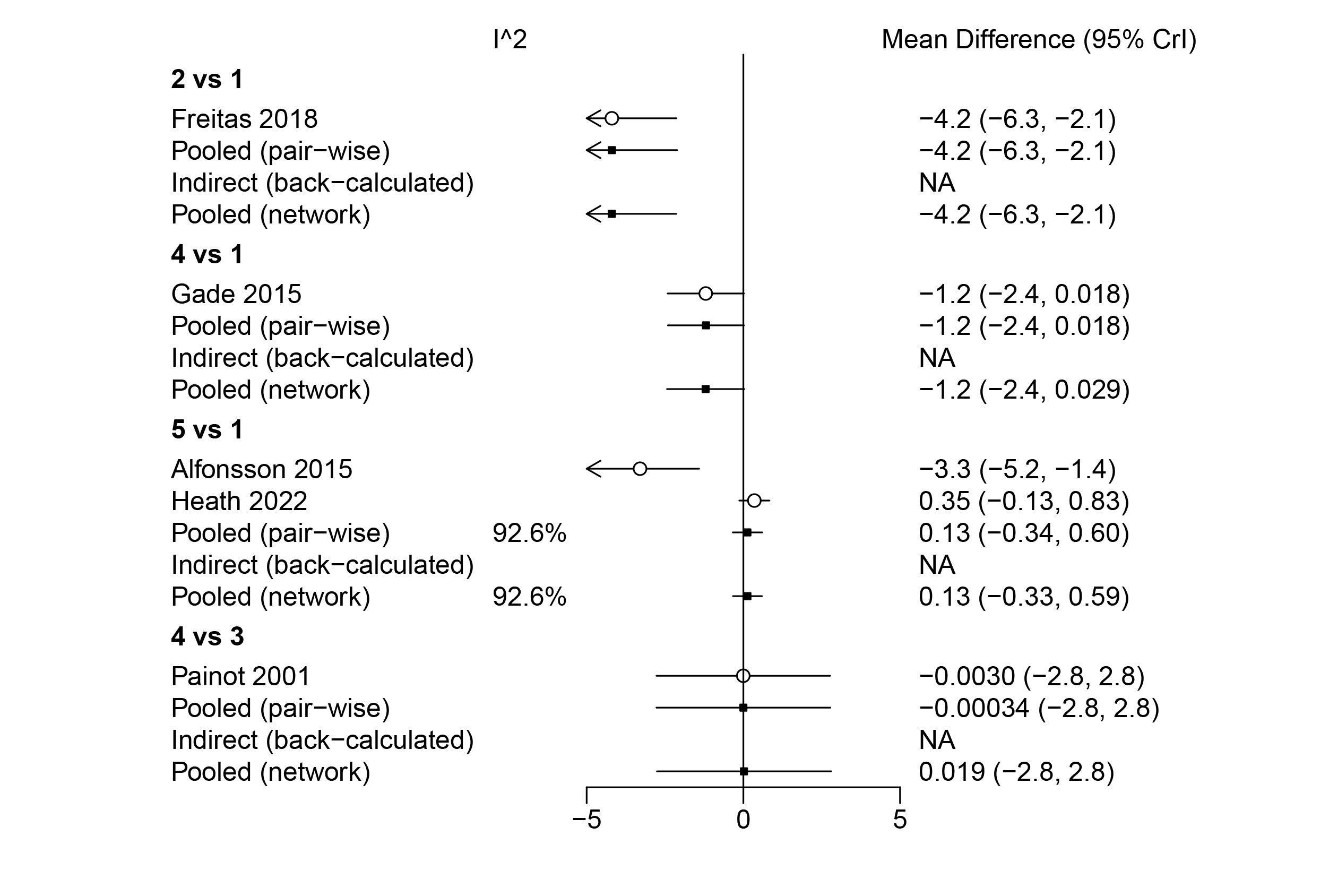


PHQ-9


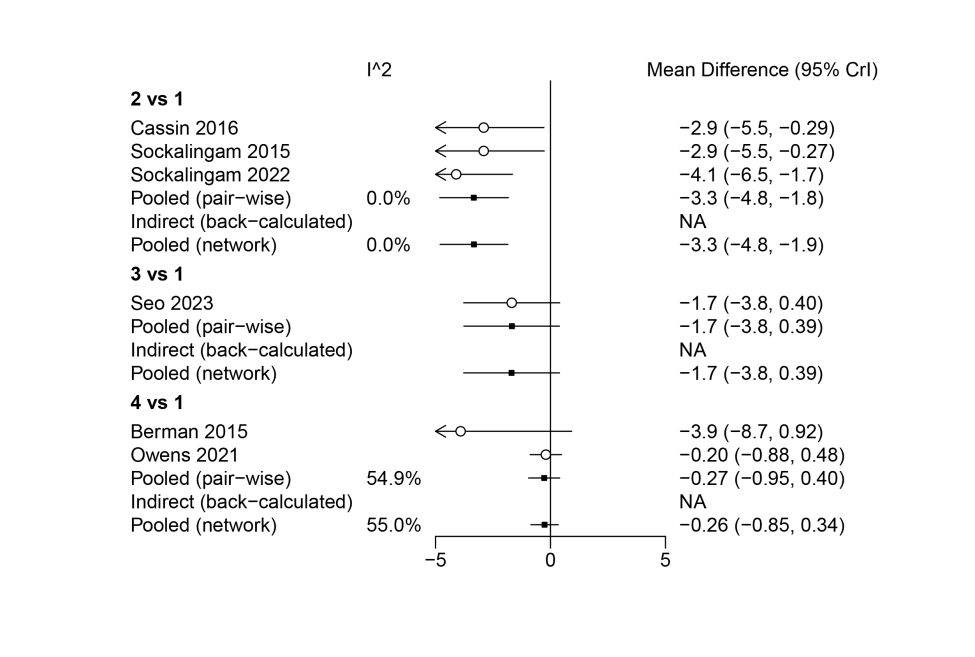

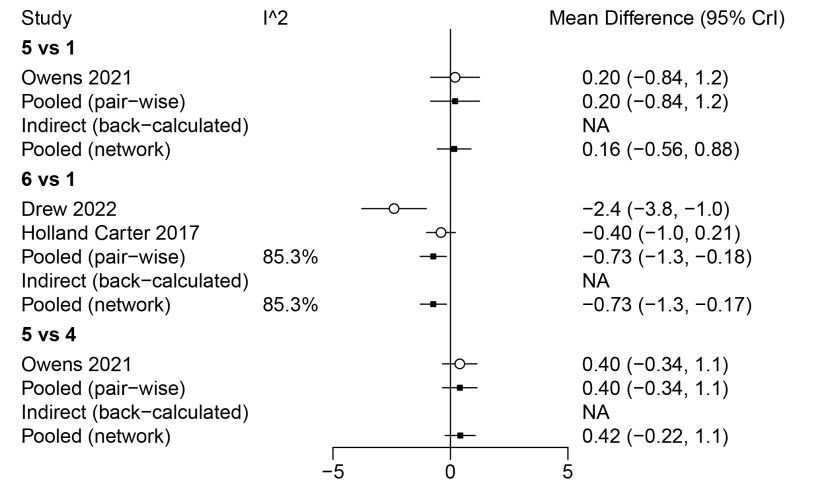


**Summary data**


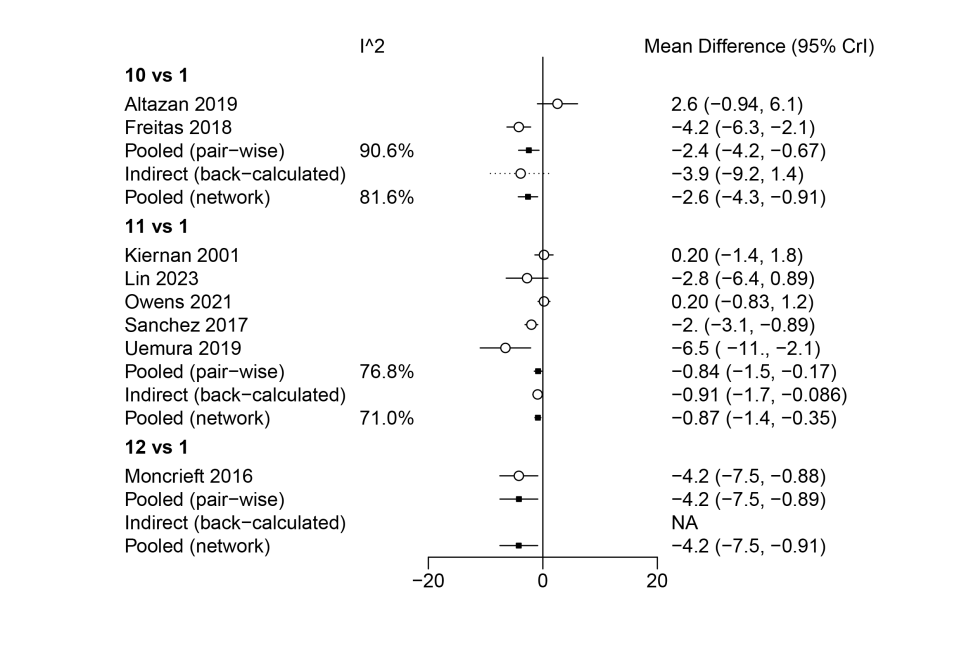

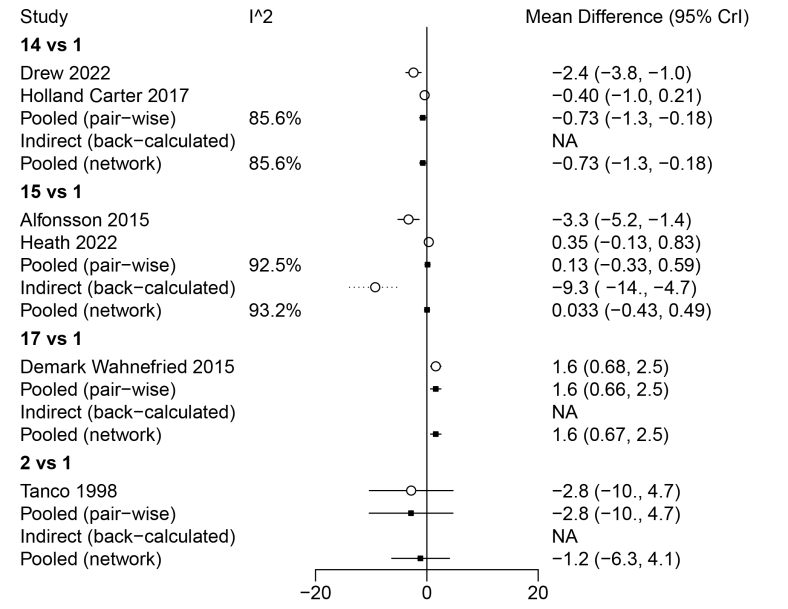


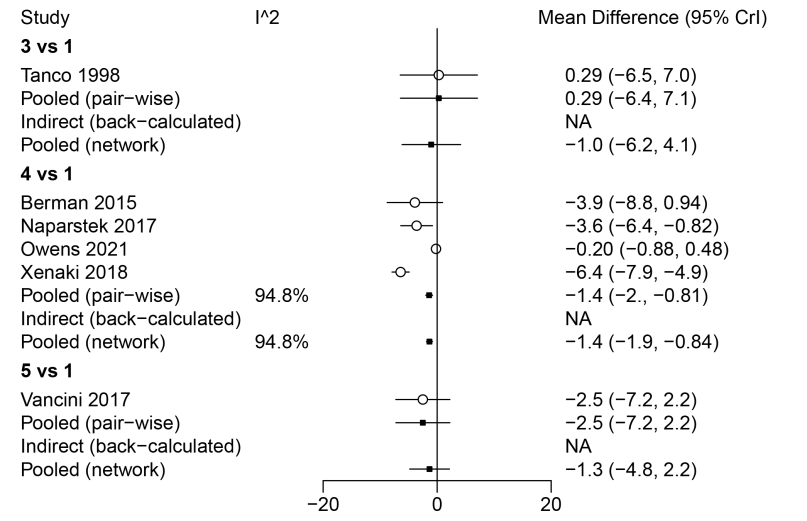

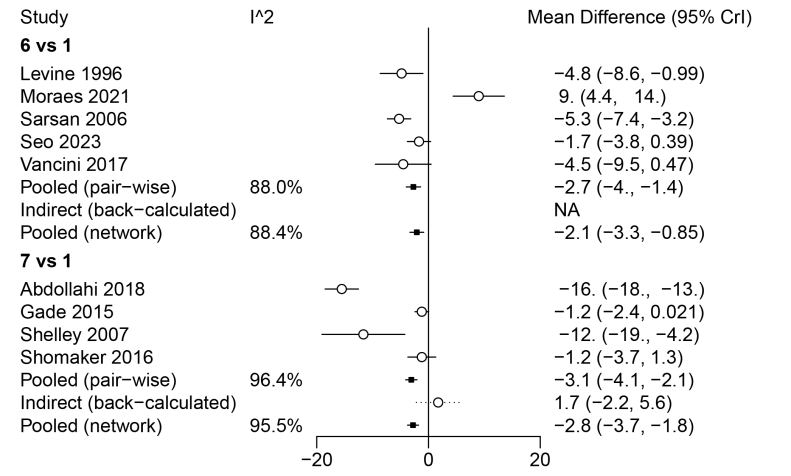


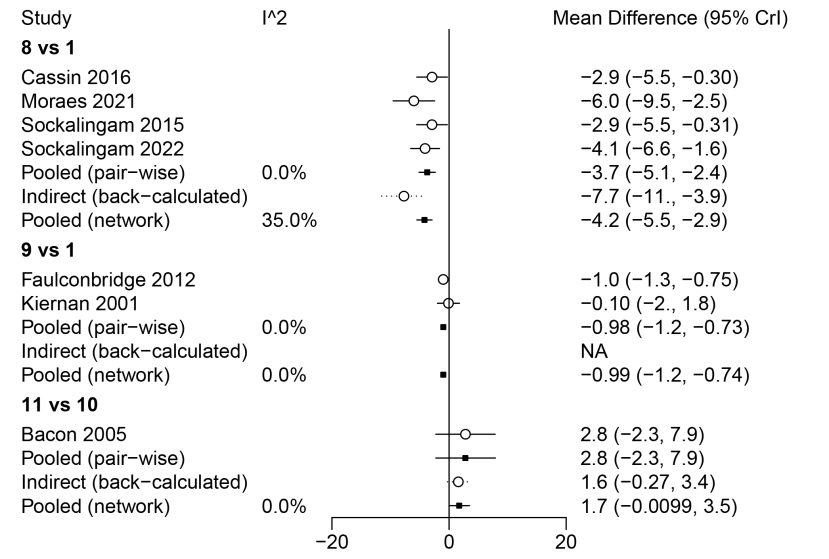

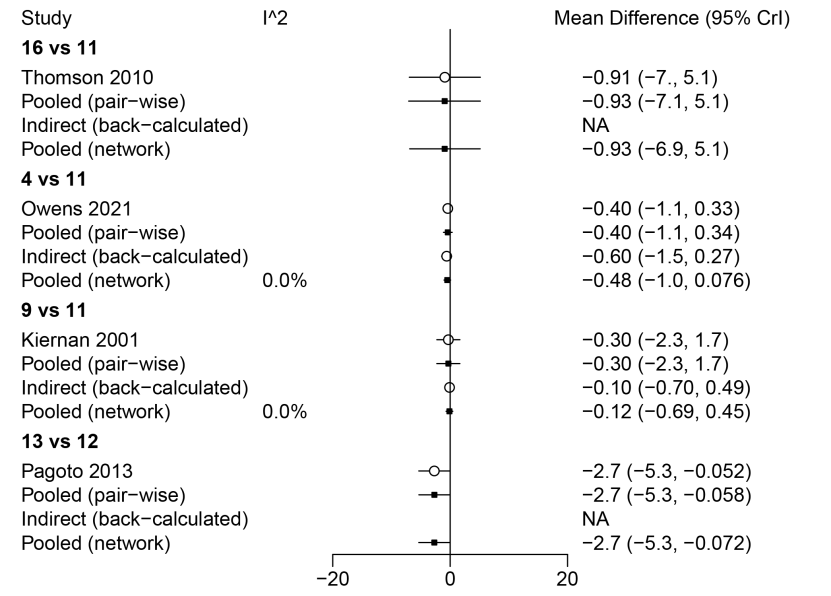


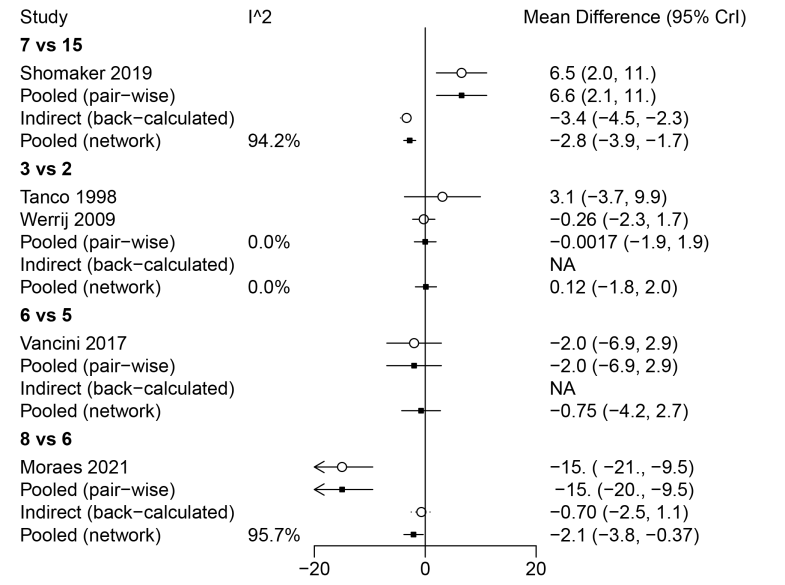


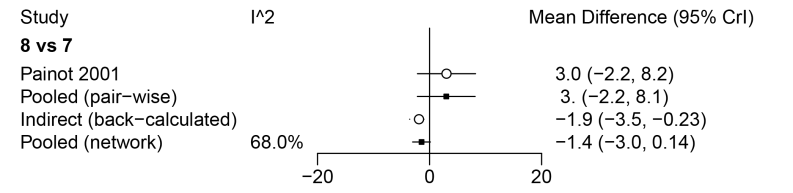

Supplement: Supplementary file 3 [file Table2.docx]

**Appendix 4** Inconsistency Test

**BDI**

**
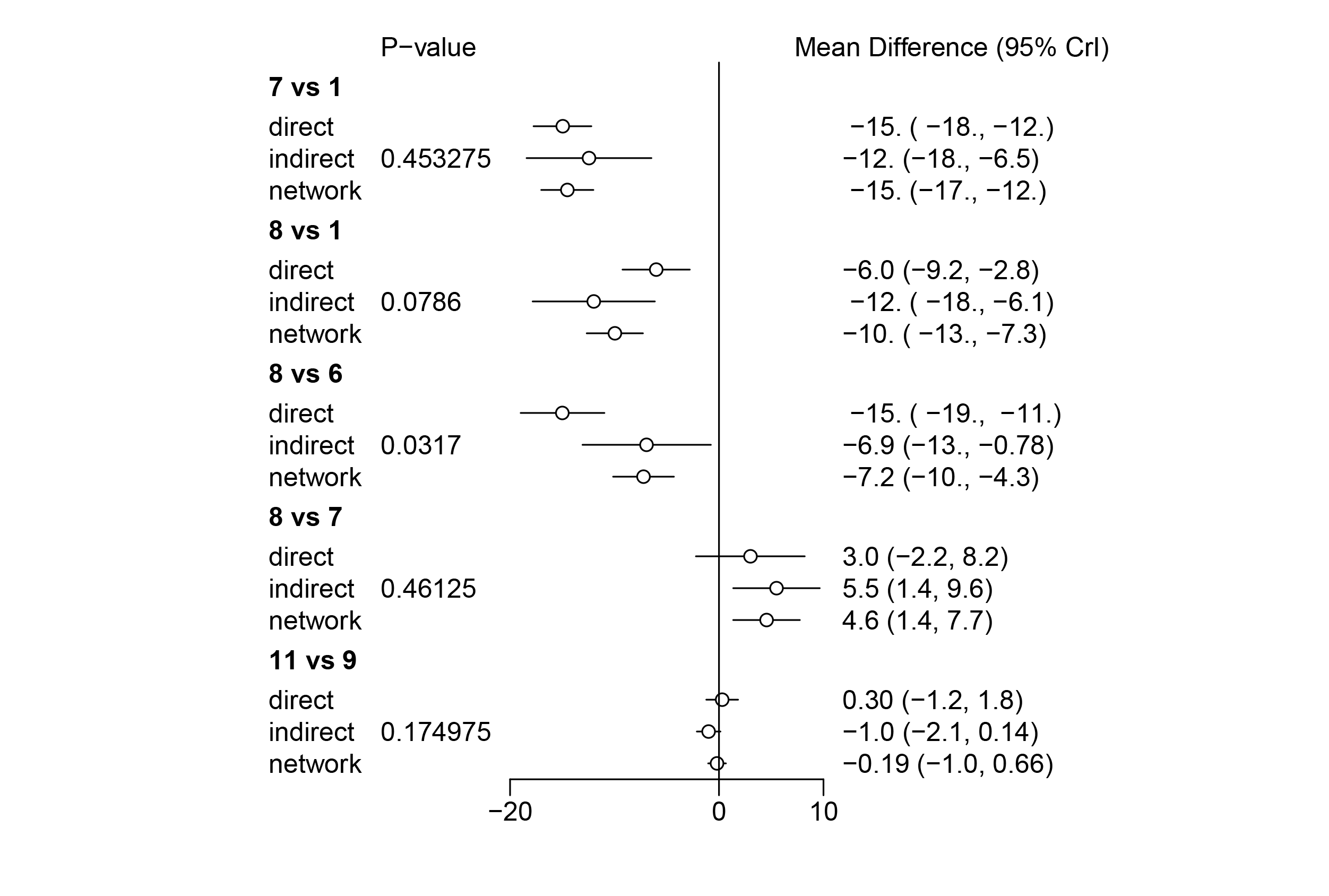
**

**Summary data**

**
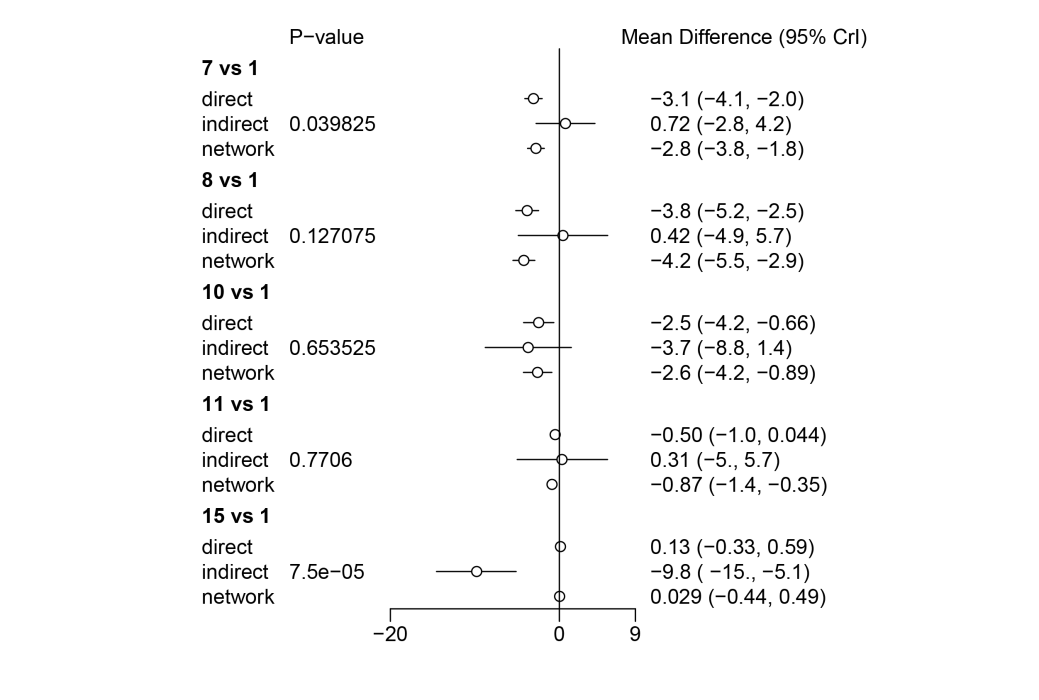

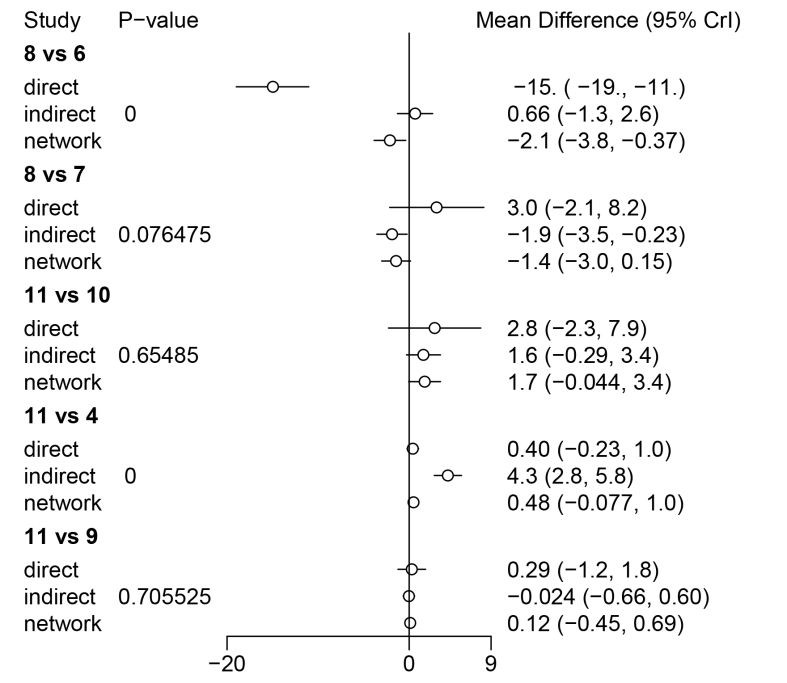
**

**
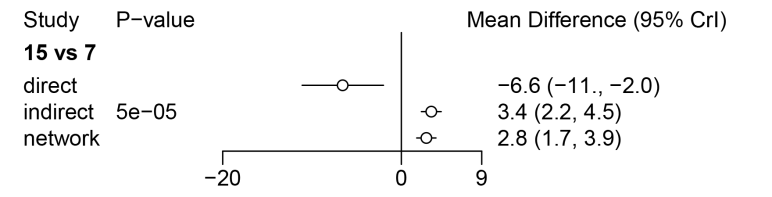
**

Supplement: Supplementary file 4 [file Table3.docx]
