# Supplementary material for: Effect of non-pharmacological interventions on depression in obese individuals: a network meta-analysis
Source: Front Psychiatry. 2026 Feb 16;17:1715475. doi: 10.3389/fpsyt.2026.1715475 (PMC12950660; doi:10.3389/fpsyt.2026.1715475)
Supplement: Supplementary file 2 [file Table1.docx]

**Appendix 1** Search Results

| Pubmed | |
| --- | --- |
| # | Query |
| 1 | Obesity[MeSH Terms] |
| 2 | Overweight[MeSH Terms] |
| 3 | "adipose tissue hyperplasia"[Title/Abstract] OR "adiposit*"[Title/Abstract] OR "corpulency"[Title/Abstract] OR "excess body weight"[Title/Abstract] OR "fat overload syndrome"[Title/Abstract] OR "obesit*"[Title/Abstract] OR "overweight"[Title/Abstract] |
| 4 | #1 OR #2 OR #3 |
| 5 | Diet[MeSH Terms] |
| 6 | "diet*"[Title/Abstract] OR "dietary effect"[Title/Abstract] OR "dietary influence"[Title/Abstract] OR "dietary survey*"[Title/Abstract] |
| 7 | Exercise[MeSH Terms] |
| 8 | "effort"[Title/Abstract] OR "exercise*"[Title/Abstract] OR "exertion"[Title/Abstract] OR "fitness training"[Title/Abstract] OR "fitness workout"[Title/Abstract] OR "human physical conditioning"[Title/Abstract] OR "physical effort"[Title/Abstract] OR "physical workout"[Title/Abstract] OR "Physical Activit*"[Title/Abstract] |
| 9 | Cognitive Behavioral Therapy[MeSH Terms] |
| 10 | "cognitive behavio*r* intervention"[Title/Abstract] OR "cognitive behavio*r* therap*"[Title/Abstract] OR "cognitive behavio*r* treatment"[Title/Abstract] OR "CBT"[Title/Abstract] OR "Cognition Therap*"[Title/Abstract] OR "Cognitive Psychotherap*"[Title/Abstract] OR "Cognitive Therap*"[Title/Abstract] |
| 11 | mindfulness-based stress reduction[MeSH Terms] |
| 12 | "mindfulness based stress reduction* "[Title/Abstract] OR "mindfulness stress reduction*"[Title/Abstract] OR "MBSR intervention"[Title/Abstract] OR "MBSR therap*"[Title/Abstract] OR "MBSR treatment"[Title/Abstract] |
| 13 | Psychosocial Intervention[MeSH Terms] |
| 14 | "psycho social therapy"[Title/Abstract] OR "psycho social treatment"[Title/Abstract] OR "Psychological Intervention*"[Title/Abstract] OR "Psychosocial Intervention*"[Title/Abstract] OR "psychosocial therapy"[Title/Abstract] OR "psychosocial treatment"[Title/Abstract] |
| 15 | Socioenvironmental Therapy[MeSH Terms] |
| 16 | "psychotherapeutic processes"[Title/Abstract] OR "psychotherapeutic training"[Title/Abstract] OR "psychotherapy"[Title/Abstract] OR "Socioenvironmental Therap*"[Title/Abstract] |
| 17 | Chronotherapy[MeSH Terms] |
| 18 | "chrono therapy"[Title/Abstract] OR "Chronotherap*"[Title/Abstract] |
| 19 | Social Support[MeSH Terms] |
| 20 | "Social Care"[Title/Abstract] OR "social support*"[Title/Abstract] |
| 21 | Yoga[MeSH Terms] |
| 22 | "yogic meditation"[Title/Abstract] OR "yoga"[Title/Abstract] |
| 23 | #5 OR #6 OR #7 OR #8 OR #9 OR #10 OR #11 OR #12 OR #13 OR #14 OR #15 OR #16 OR #17 OR #18 OR #19 OR #20 OR #21 OR #22 |
| 24 | Depression[MeSH Terms] |
| 25 | "depression"[Title/Abstract] OR "depressive disease"[Title/Abstract] OR "depressive disorder"[Title/Abstract] OR "depressive episode"[Title/Abstract] OR "depressive illness"[Title/Abstract] OR "depressive personality disorder"[Title/Abstract] OR "depressive state"[Title/Abstract] OR "depressive symptom*"[Title/Abstract] OR "depressive syndrome"[Title/Abstract] OR "depressivity"[Title/Abstract] |
| 26 | #24 OR #25 |
| 27 | random*[Title/Abstract] |
| 28 | #4 AND #23 AND #26 AND #27 |
| 29 | Cited In for PMID: 30340503 |

| Embase | |
| --- | --- |
| # | Query |
| 1 | 'obesity'/exp |
| 2 | 'adipose tissue hyperplasia':ti,ab,kw OR 'adiposit*':ti,ab,kw OR 'corpulency':ti,ab,kw OR 'excess body weight':ti,ab,kw OR 'fat overload syndrome':ti,ab,kw OR 'obesit*':ti,ab,kw OR 'overweight':ti,ab,kw |
| 3 | #1 OR #2 |
| 4 | 'diet'/exp |
| 5 | 'diet*':ti,ab,kw OR 'dietary effect':ti,ab,kw OR 'dietary influence':ti,ab,kw OR 'dietary survey*':ti,ab,kw |
| 6 | 'exercise'/exp |
| 7 | 'effort':ti,ab,kw OR 'exercise*':ti,ab,kw OR 'exertion':ti,ab,kw OR 'fitness training':ti,ab,kw OR 'fitness workout':ti,ab,kw OR 'human physical conditioning':ti,ab,kw OR 'physical effort':ti,ab,kw OR 'physical workout':ti,ab,kw OR 'physical activit*':ti,ab,kw |
| 8 | 'cognitive behavioral therapy'/exp |
| 9 | 'cognitive behavio*r* intervention':ti,ab,kw OR 'cognitive behavio*r* therap*':ti,ab,kw OR 'cognitive behavio*r* treatment':ti,ab,kw OR 'cbt':ti,ab,kw OR 'cognition therap*':ti,ab,kw OR 'cognitive psychotherap*':ti,ab,kw OR 'cognitive therap*':ti,ab,kw |
| 10 | 'mindfulness-based stress reduction'/exp |
| 11 | 'mindfulness based stress reduction*':ti,ab,kw OR 'mindfulness stress reduction*':ti,ab,kw OR 'mbsr intervention':ti,ab,kw OR 'mbsr therap*':ti,ab,kw OR 'mbsr treatment':ti,ab,kw |
| 12 | 'psychosocial intervention'/exp |
| 13 | 'psycho social therapy':ti,ab,kw OR 'psycho social treatment':ti,ab,kw OR 'psychological intervention*':ti,ab,kw OR 'psychosocial intervention*':ti,ab,kw OR 'psychosocial therapy':ti,ab,kw OR 'psychosocial treatment':ti,ab,kw |
| 14 | 'psychotherapy'/exp |
| 15 | 'psychotherapeutic processes':ti,ab,kw OR 'psychotherapeutic training':ti,ab,kw OR 'psychotherapy':ti,ab,kw OR 'socioenvironmental therap*':ti,ab,kw |
| 16 | 'chronotherapy'/exp |
| 17 | 'chrono therapy':ti,ab,kw OR 'chronotherap*':ti,ab,kw |
| 18 | 'social support'/exp |
| 19 | 'social care':ti,ab,kw OR 'social support*':ti,ab,kw |
| 20 | 'yoga'/exp |
| 21 | 'yogic meditation':ti,ab,kw OR 'yoga':ti,ab,kw |
| 22 | #4 OR #5 OR #6 OR #7 OR #8 OR #9 OR #10 OR #11 OR #12 OR #13 OR #14 OR #15 OR #16 OR #17 OR #18 OR #19 OR #20 OR #21 |
| 23 | 'depression'/exp |
| 24 | 'depression':ti,ab,kw OR 'depressive disease':ti,ab,kw OR 'depressive disorder':ti,ab,kw OR 'depressive episode':ti,ab,kw OR 'depressive illness':ti,ab,kw OR 'depressive personality disorder':ti,ab,kw OR 'depressive state':ti,ab,kw OR 'depressive symptom*':ti,ab,kw OR 'depressive syndrome':ti,ab,kw OR 'depressivity':ti,ab,kw |
| 25 | #23 OR #24 |
| 26 | random*:ti,ab,kw |
| 27 | #3 AND #22 AND #25 AND #26 |

| Cochrane Library | |
| --- | --- |
| # | Query |
| 1 | MeSH descriptor: [Obesity] explode all trees |
| 2 | MeSH descriptor: [Overweight] explode all trees |
| 3 | ('adipose tissue hyperplasia' OR 'adiposit*' OR 'corpulency' OR 'excess body weight' OR 'fat overload syndrome' OR 'obesit*' OR 'overweight'):ti,ab,kw |
| 4 | #1 OR #2 OR #3 |
| 5 | MeSH descriptor: [Diet] explode all trees |
| 6 | ('diet*' OR 'dietary effect' OR 'dietary influence' OR 'dietary survey*'):ti,ab,kw |
| 7 | MeSH descriptor: [Exercise] explode all trees |
| 8 | ('effort' OR 'exercise*' OR 'exertion' OR 'fitness training' OR 'fitness workout' OR 'human physical conditioning' OR 'physical effort' OR 'physical workout' OR 'Physical Activit*'):ti,ab,kw |
| 9 | MeSH descriptor: [Cognitive Behavioral Therapy] explode all trees |
| 10 | ('cognitive behavio*r* intervention' OR 'cognitive behavio*r* therap*' OR 'cognitive behavio*r* treatment' OR 'CBT' OR 'Cognition Therap*' OR 'Cognitive Psychotherap*' OR 'Cognitive Therap*'):ti,ab,kw |
| 11 | MeSH descriptor: [Mindfulness-Based Stress Reduction] explode all trees |
| 12 | ('mindfulness based stress reduction* ' OR 'mindfulness stress reduction*' OR 'MBSR intervention' OR 'MBSR therap*' OR 'MBSR treatment'):ti,ab,kw |
| 13 | MeSH descriptor: [Psychosocial Intervention] explode all trees |
| 14 | ('psycho social therapy' OR 'psycho social treatment' OR 'Psychological Intervention*' OR 'Psychosocial Intervention*' OR 'psychosocial therapy' OR 'psychosocial treatment'):ti,ab,kw |
| 15 | MeSH descriptor: [Socioenvironmental Therapy] explode all trees |
| 16 | ('psychotherapeutic processes' OR 'psychotherapeutic training' OR 'psychotherapy' OR 'Socioenvironmental Therap*'):ti,ab,kw |
| 17 | MeSH descriptor: [Chronotherapy] explode all trees |
| 18 | ('chrono therapy' OR 'Chronotherap*'):ti,ab,kw |
| 19 | MeSH descriptor: [Social Support] explode all trees |
| 20 | ('Social Care' OR 'social support*'):ti,ab,kw |
| 21 | MeSH descriptor: [Yoga] explode all trees |
| 22 | ('yogic meditation' OR 'yoga'):ti,ab,kw |
| 23 | #5 OR #6 OR #7 OR #8 OR #9 OR #10 OR #11 OR #12 OR #13 OR #14 OR #15 OR #16 OR #17 OR #18 OR #19 OR #20 OR #21 OR #22 |
| 24 | MeSH descriptor: [Depression] explode all trees |
| 25 | ('depression' OR 'depressive disease' OR 'depressive disorder' OR 'depressive episode' OR 'depressive illness' OR 'depressive personality disorder' OR 'depressive state' OR 'depressive symptom*' OR 'depressive syndrome' OR 'depressivity'):ti,ab,kw |
| 26 | #24 OR #25 |
| 27 | (random*):ti,ab,kw |
| 28 | #4 AND #23 AND #26 AND #27 |

| Web of Science | |
| --- | --- |
| # | Query |
| 1 | TS=((adipose tissue hyperplasia) OR (adiposit*) OR (corpulency) OR (excess body weight) OR (fat overload syndrome) OR (obesit*) OR (overweight)) |
| 2 | TS=((diet*) OR (dietary effect) OR (dietary influence) OR (dietary survey*)) |
| 3 | TS=((effort) OR (exercise*) OR (exertion) OR (fitness training) OR (fitness workout) OR (human physical conditioning) OR (physical effort) OR (physical workout) OR (Physical Activit*)) |
| 4 | TS=((cognitive behavio*r* intervention) OR (cognitive behavio*r* therap*) OR (cognitive behavio*r* treatment) OR (CBT) OR (Cognition Therap*) OR (Cognitive Psychotherap*) OR (Cognitive Therap*)) |
| 5 | TS=((mindfulness based stress reduction* ) OR (mindfulness stress reduction*) OR (MBSR intervention) OR (MBSR therap*) OR (MBSR treatment)) |
| 6 | TS=((psycho social therapy) OR (psycho social treatment) OR (Psychological Intervention*) OR (Psychosocial Intervention*) OR (psychosocial therapy) OR (psychosocial treatment)) |
| 7 | TS=((psychotherapeutic processes) OR (psychotherapeutic training) OR (psychotherapy) OR (Socioenvironmental Therap*)) |
| 8 | TS=((chrono therapy) OR (Chronotherap*)) |
| 9 | TS=((Social Care) OR (social support*)) |
| 10 | TS=((yogic meditation) OR (yoga)) |
| 11 | #10 OR #9 OR #8 OR #7 OR #6 OR #5 OR #4 OR #3 OR #2 |
| 12 | TS=((depression) OR (depressive disease) OR (depressive disorder) OR (depressive episode) OR (depressive illness) OR (depressive personality disorder) OR (depressive state) OR (depressive symptom*) OR (depressive syndrome) OR (depressivity)) |
| 13 | TS=(random*) |
| 14 | #1 AND #11 AND #12 AND #13 |
