# Supplementary material for: Effect of non-pharmacological interventions on depression in obese individuals: a network meta-analysis
Source: Front Psychiatry. 2026 Feb 16;17:1715475. doi: 10.3389/fpsyt.2026.1715475 (PMC12950660; doi:10.3389/fpsyt.2026.1715475)
Supplement: Supplementary file 5 [file Table4.docx]

**Appendix 5** Sensitivity Analysis

**SUCRA for CES-D**


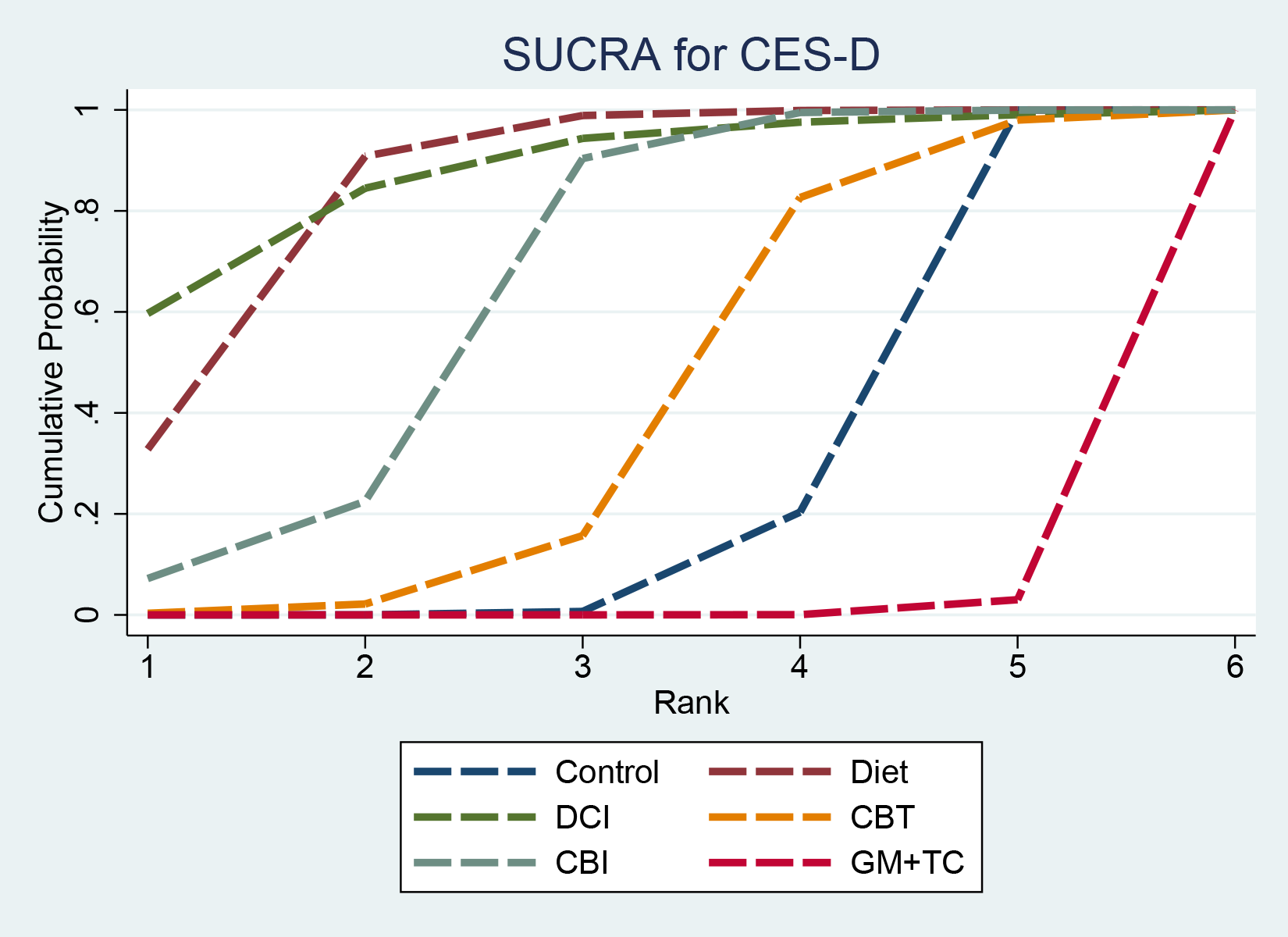


**SUCRA for Summary data**


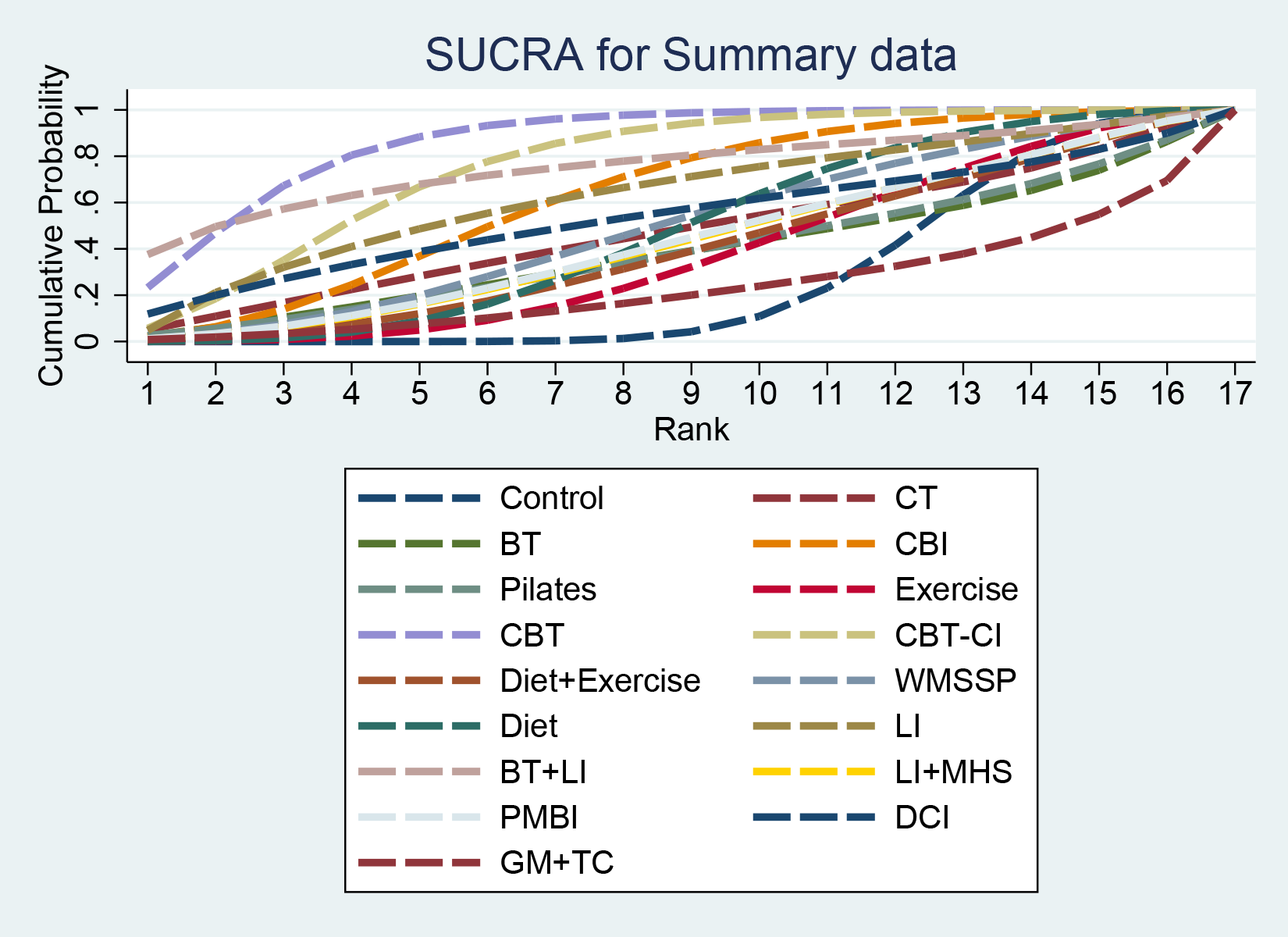


**League table for CES-D after excluding the study by Shomaker.2019**

| No. | X1 | X2 | X3 | X4 | X5 | X6 |
| --- | --- | --- | --- | --- | --- | --- |
| 1 | 1 | -6.54 (-10.94, -2.12) | -7.5 (-14.96, 0.05) | -1.2 (-3.72, 1.32) | -3.59 (-6.38, -0.82) | 1.6 (0.68, 2.53) |
| 2 | 6.54 (2.12, 10.94) | 2 | -0.94 (-6.95, 5.15) | 5.34 (0.25, 10.42) | 2.95 (-2.27, 8.15) | 8.14 (3.63, 12.62) |
| 3 | 7.5 (-0.05, 14.96) | 0.94 (-5.15, 6.95) | 3 | 6.29 (-1.64, 14.18) | 3.89 (-4.13, 11.86) | 9.11 (1.49, 16.61) |
| 4 | 1.2 (-1.32, 3.72) | -5.34 (-10.42, -0.25) | -6.29 (-14.18, 1.64) | 4 | -2.4 (-6.15, 1.36) | 2.81 (0.12, 5.49) |
| 5 | 3.59 (0.82, 6.38) | -2.95 (-8.15, 2.27) | -3.89 (-11.86, 4.13) | 2.4 (-1.36, 6.15) | 5 | 5.2 (2.27, 8.14) |
| 6 | -1.6 (-2.53, -0.68) | -8.14 (-12.62, -3.63) | -9.11 (-16.61, -1.49) | -2.81 (-5.49, -0.12) | -5.2 (-8.14, -2.27) | 6 |

**League table for summary data after excluding the study by Shomaker.2019**

| No. | **X1** | **X2** | **X3** | **X4** | **X5** | **X6** | **X7** | **X8** | **X9** | **X10** | **X11** | **X12** | **X13** | **X14** | **X15** | **X16** | **X17** |
| --- | --- | --- | --- | --- | --- | --- | --- | --- | --- | --- | --- | --- | --- | --- | --- | --- | --- |
| 1 | 1 | -1.73 (-11.11, 7.67) | -0.63 (-9.94, 8.78) | -3.56 (-7.84, 0.68) | -0.73 (-8.94, 7.56) | -0.89 (-4.81, 3.06) | -6.94 (-11.17, -2.8) | -5.19 (-9.16, -1.19) | -1.07 (-6.69, 4.53) | -2.08 (-7.48, 3.24) | -1.86 (-5.48, 1.71) | -4.17 (-13.19, 4.79) | -6.92 (-19.47, 5.75) | -1.39 (-7.38, 4.59) | -1.44 (-7.46, 4.6) | -2.79 (-13.55, 8) | 1.62 (-6.79, 10.02) |
| 2 | 1.73 (-7.67, 11.11) | 2 | 1.13 (-5.4, 7.69) | -1.81 (-12.14, 8.4) | 1.01 (-11.6, 13.58) | 0.85 (-9.34, 11.02) | -5.21 (-15.58, 4.94) | -3.45 (-13.67, 6.73) | 0.67 (-10.3, 11.61) | -0.36 (-11.22, 10.46) | -0.15 (-10.25, 9.96) | -2.45 (-15.43, 10.47) | -5.17 (-20.82, 10.51) | 0.32 (-10.84, 11.53) | 0.31 (-10.87, 11.43) | -1.11 (-15.32, 13.44) | 3.36 (-9.3, 16) |
| 3 | 0.63 (-8.78, 9.94) | -1.13 (-7.69, 5.4) | 3 | -2.93 (-13.32, 7.28) | -0.07 (-12.68, 12.38) | -0.27 (-10.43, 9.77) | -6.32 (-16.73, 3.8) | -4.56 (-14.79, 5.54) | -0.47 (-11.5, 10.38) | -1.47 (-12.27, 9.31) | -1.26 (-11.35, 8.74) | -3.57 (-16.57, 9.29) | -6.29 (-21.94, 9.29) | -0.79 (-11.88, 10.35) | -0.81 (-11.92, 10.18) | -2.22 (-16.36, 12.1) | 2.19 (-10.38, 14.8) |
| 4 | 3.56 (-0.68, 7.84) | 1.81 (-8.4, 12.14) | 2.93 (-7.28, 13.32) | 4 | 2.83 (-6.44, 12.17) | 2.66 (-3.12, 8.49) | -3.37 (-9.38, 2.54) | -1.62 (-7.49, 4.21) | 2.48 (-4.48, 9.46) | 1.46 (-5.29, 8.22) | 1.7 (-3.48, 6.86) | -0.62 (-10.57, 9.34) | -3.37 (-16.69, 9.99) | 2.15 (-5.17, 9.53) | 2.12 (-5.29, 9.49) | 0.77 (-10.68, 12.21) | 5.19 (-4.24, 14.57) |
| 5 | 0.73 (-7.56, 8.94) | -1.01 (-13.58, 11.6) | 0.07 (-12.38, 12.68) | -2.83 (-12.17, 6.44) | 5 | -0.17 (-8.44, 8.11) | -6.2 (-15.62, 2.97) | -4.45 (-13.58, 4.57) | -0.33 (-10.39, 9.56) | -1.36 (-11.26, 8.43) | -1.14 (-10.23, 7.81) | -3.44 (-15.67, 8.63) | -6.2 (-21.24, 8.79) | -0.67 (-10.88, 9.52) | -0.72 (-10.97, 9.46) | -2.08 (-15.67, 11.62) | 2.33 (-9.42, 14.1) |
| 6 | 0.89 (-3.06, 4.81) | -0.85 (-11.02, 9.34) | 0.27 (-9.77, 10.43) | -2.66 (-8.49, 3.12) | 0.17 (-8.11, 8.44) | 6 | -6.04 (-11.82, -0.43) | -4.29 (-9.65, 0.99) | -0.17 (-7.05, 6.64) | -1.18 (-7.94, 5.4) | -0.95 (-6.36, 4.3) | -3.28 (-13.17, 6.5) | -6.01 (-19.27, 7.16) | -0.5 (-7.67, 6.6) | -0.53 (-7.79, 6.66) | -1.9 (-13.45, 9.59) | 2.53 (-6.88, 11.7) |
| 7 | 6.94 (2.8, 11.17) | 5.21 (-4.94, 15.58) | 6.32 (-3.8, 16.73) | 3.37 (-2.54, 9.38) | 6.2 (-2.97, 15.62) | 6.04 (0.43, 11.82) | 7 | 1.75 (-3.43, 7.12) | 5.87 (-1.12, 12.92) | 4.85 (-1.91, 11.7) | 5.06 (-0.37, 10.64) | 2.75 (-7.1, 12.71) | 0.02 (-13.22, 13.4) | 5.52 (-1.71, 12.9) | 5.5 (-1.75, 12.93) | 4.1 (-7.3, 15.82) | 8.54 (-0.79, 17.97) |
| 8 | 5.19 (1.19, 9.16) | 3.45 (-6.73, 13.67) | 4.56 (-5.54, 14.79) | 1.62 (-4.21, 7.49) | 4.45 (-4.57, 13.58) | 4.29 (-0.99, 9.65) | -1.75 (-7.12, 3.43) | 8 | 4.12 (-2.78, 10.97) | 3.1 (-3.6, 9.76) | 3.31 (-2.06, 8.69) | 0.98 (-8.84, 10.82) | -1.72 (-15.02, 11.51) | 3.77 (-3.38, 11.02) | 3.76 (-3.5, 10.93) | 2.39 (-9.07, 14) | 6.82 (-2.46, 16.07) |
| 9 | 1.07 (-4.53, 6.69) | -0.67 (-11.61, 10.3) | 0.47 (-10.38, 11.5) | -2.48 (-9.46, 4.48) | 0.33 (-9.56, 10.39) | 0.17 (-6.64, 7.05) | -5.87 (-12.92, 1.12) | -4.12 (-10.97, 2.78) | 9 | -1.01 (-8.64, 6.62) | -0.8 (-6.93, 5.26) | -3.12 (-13.77, 7.51) | -5.85 (-19.62, 8.02) | -0.32 (-8.52, 7.9) | -0.38 (-8.63, 7.88) | -1.7 (-13.61, 10.13) | 2.69 (-7.39, 12.77) |
| 10 | 2.08 (-3.24, 7.48) | 0.36 (-10.46, 11.22) | 1.47 (-9.31, 12.27) | -1.46 (-8.22, 5.29) | 1.36 (-8.43, 11.26) | 1.18 (-5.4, 7.94) | -4.85 (-11.7, 1.91) | -3.1 (-9.76, 3.6) | 1.01 (-6.62, 8.64) | 10 | 0.22 (-5.62, 6.01) | -2.09 (-12.6, 8.4) | -4.82 (-18.44, 8.87) | 0.68 (-7.37, 8.71) | 0.66 (-7.39, 8.64) | -0.71 (-12.42, 11.08) | 3.71 (-6.27, 13.61) |
| 11 | 1.86 (-1.71, 5.48) | 0.15 (-9.96, 10.25) | 1.26 (-8.74, 11.35) | -1.7 (-6.86, 3.48) | 1.14 (-7.81, 10.23) | 0.95 (-4.3, 6.36) | -5.06 (-10.64, 0.37) | -3.31 (-8.69, 2.06) | 0.8 (-5.26, 6.93) | -0.22 (-6.01, 5.62) | 11 | -2.31 (-12.01, 7.25) | -5.03 (-18.16, 8.14) | 0.47 (-6.49, 7.5) | 0.44 (-6.6, 7.37) | -0.95 (-11.09, 9.32) | 3.49 (-5.66, 12.66) |
| 12 | 4.17 (-4.79, 13.19) | 2.45 (-10.47, 15.43) | 3.57 (-9.29, 16.57) | 0.62 (-9.34, 10.57) | 3.44 (-8.63, 15.67) | 3.28 (-6.5, 13.17) | -2.75 (-12.71, 7.1) | -0.98 (-10.82, 8.84) | 3.12 (-7.51, 13.77) | 2.09 (-8.4, 12.6) | 2.31 (-7.25, 12.01) | 12 | -2.73 (-11.52, 6.07) | 2.78 (-8, 13.6) | 2.74 (-7.97, 13.59) | 1.39 (-12.52, 15.5) | 5.79 (-6.48, 18.16) |
| 13 | 6.92 (-5.75, 19.47) | 5.17 (-10.51, 20.82) | 6.29 (-9.29, 21.94) | 3.37 (-9.99, 16.69) | 6.2 (-8.79, 21.24) | 6.01 (-7.16, 19.27) | -0.02 (-13.4, 13.22) | 1.72 (-11.51, 15.02) | 5.85 (-8.02, 19.62) | 4.82 (-8.87, 18.44) | 5.03 (-8.14, 18.16) | 2.73 (-6.07, 11.52) | 13 | 5.51 (-8.43, 19.53) | 5.45 (-8.45, 19.47) | 4.1 (-12.48, 20.72) | 8.5 (-6.51, 23.71) |
| 14 | 1.39 (-4.59, 7.38) | -0.32 (-11.53, 10.84) | 0.79 (-10.35, 11.88) | -2.15 (-9.53, 5.17) | 0.67 (-9.52, 10.88) | 0.5 (-6.6, 7.67) | -5.52 (-12.9, 1.71) | -3.77 (-11.02, 3.38) | 0.32 (-7.9, 8.52) | -0.68 (-8.71, 7.37) | -0.47 (-7.5, 6.49) | -2.78 (-13.6, 8) | -5.51 (-19.53, 8.43) | 14 | -0.04 (-8.57, 8.42) | -1.44 (-13.71, 10.98) | 3.02 (-7.33, 13.36) |
| 15 | 1.44 (-4.6, 7.46) | -0.31 (-11.43, 10.87) | 0.81 (-10.18, 11.92) | -2.12 (-9.49, 5.29) | 0.72 (-9.46, 10.97) | 0.53 (-6.66, 7.79) | -5.5 (-12.93, 1.75) | -3.76 (-10.93, 3.5) | 0.38 (-7.88, 8.63) | -0.66 (-8.64, 7.39) | -0.44 (-7.37, 6.6) | -2.74 (-13.59, 7.97) | -5.45 (-19.47, 8.45) | 0.04 (-8.42, 8.57) | 15 | -1.38 (-13.75, 11) | 3.04 (-7.28, 13.3) |
| 16 | 2.79 (-8, 13.55) | 1.11 (-13.44, 15.32) | 2.22 (-12.1, 16.36) | -0.77 (-12.21, 10.68) | 2.08 (-11.62, 15.67) | 1.9 (-9.59, 13.45) | -4.1 (-15.82, 7.3) | -2.39 (-14, 9.07) | 1.7 (-10.13, 13.61) | 0.71 (-11.08, 12.42) | 0.95 (-9.32, 11.09) | -1.39 (-15.5, 12.52) | -4.1 (-20.72, 12.48) | 1.44 (-10.98, 13.71) | 1.38 (-11, 13.75) | 16 | 4.37 (-9.25, 18.03) |
| 17 | -1.62 (-10.02, 6.79) | -3.36 (-16, 9.3) | -2.19 (-14.8, 10.38) | -5.19 (-14.57, 4.24) | -2.33 (-14.1, 9.42) | -2.53 (-11.7, 6.88) | -8.54 (-17.97, 0.79) | -6.82 (-16.07, 2.46) | -2.69 (-12.77, 7.39) | -3.71 (-13.61, 6.27) | -3.49 (-12.66, 5.66) | -5.79 (-18.16, 6.48) | -8.5 (-23.71, 6.51) | -3.02 (-13.36, 7.33) | -3.04 (-13.3, 7.28) | -4.37 (-18.03, 9.25) | 17 |
